# Angiopoetin-2 Signals Do Not Mediate the Hypervascularization of Islets in Type 2 Diabetes

**DOI:** 10.1371/journal.pone.0161834

**Published:** 2016-09-12

**Authors:** Payal Shah, Navina Lueschen, Amin Ardestani, Jose Oberholzer, Johan Olerud, Per-Ola Carlsson, Kathrin Maedler

**Affiliations:** 1 Centre for Biomolecular Interactions, University of Bremen, Bremen, Germany; 2 Division of Transplantation, University of Illinois at Chicago, Chicago, Illinois, United States of America; 3 Department of Immunology, Genetics and pathology, Uppsala University, Uppsala, Sweden; 4 Department of Medical cell biology and Department of Medical Sciences, Uppsala University, Uppsala, Sweden; 5 German Center for Diabetes Research (DZD) project partner, University of Bremen, Bremen, Germany; Institut d'Investigacions Biomèdiques August Pi i Sunyer, SPAIN

## Abstract

**Aims:**

Changes in the islet vasculature have been implicated in the regulation of β-cell survival and function during the progression to type 2 diabetes (T2D). Failure of the β-cell to compensate for the increased insulin demand in obesity eventually leads to diabetes; as a result of the complex interplay of genetic and environmental factors (e.g. ongoing inflammation within the islets) and impaired vascular function. The Angiopoietin/Tie (Ang/Tie) angiogenic system maintains vasculature and is closely related to organ inflammation and angiogenesis. In this study we aimed to identify whether the vessel area within the islets changes in diabetes and whether such changes would be triggered by the Tie-antagonist Ang-2.

**Methods:**

Immunohistochemical and qPCR analyses to follow islet vascularization and Ang/Tie levels were performed in human pancreatic autopsies and isolated human and mouse islets. The effect of Ang-2 was assessed in β-cell-specific Ang-2 overexpressing mice during high fat diet (HFD) feeding.

**Results:**

Islet vessel area was increased in autopsy pancreases from patients with T2D. The vessel markers Tie-1, Tie-2 and CD31 were upregulated in mouse islets upon HFD feeding from 8 to 24 weeks. Ang-2 was transiently upregulated in mouse islets at 8 weeks of HFD and under glucolipotoxic conditions (22.2 mM glucose/ 0.5 mM palmitate) *in vitro* in human and mouse islets, in contrast to its downregulation by cytokines (IL-1β, IFN-ɣ and TNF-α). Ang-1 on the other hand was oppositely regulated, with a significant loss under glucolipotoxic condition, a trend to reduce in islets from patients with T2D and an upregulation by cytokines. Modulation of such changes in Ang-2 by its overexpression or the inhibition of its receptor Tie-2 impaired β-cell function at basal conditions but protected islets from cytokine induced apoptosis. *In vivo*, β-cell-specific Ang-2 overexpression in mice induced hypervascularization under normal diet but contrastingly led to hypovascularized islets in response to HFD together with increased apoptosis and reduced β-cell mass.

**Conclusions:**

Islet hypervascularization occurs in T2D. A balanced expression of the Ang1/Ang2 system is important for islet physiology. Ang-2 prevents β-cell mass and islet vascular adaptation in response to HFD feeding with no major influence on glucose homeostasis.

**Highlights**

Islet vessel area is increased in autopsy pancreases from patients with T2D.

Modulation of Ang-2 has ambiguous effects; its overexpression is only protective under cytotoxic conditions, where it is pathophysiologically downregulated.

A physiological equilibrium of Ang-2/ Tie-2 signaling under diabetic conditions is important for maintaining β-cell survival and function.

## Introduction

Pancreatic islets are densely vascularized mini-organs constituting 1–2% of the pancreatic mass but supplied by 5–15% of the pancreatic blood flow [1, 2]. The islets are penetrated by a specialized endothelium with more highly fenestrated capillaries than the exocrine tissue and with a unique basement membrane [3]. The laminin isoforms are known to directly influence β-cell function and proliferation [4]. In their remarkable study Lammert *et al*. showed that the endothelium sends inductive signals to direct islet development, beyond merely serving the metabolic demand [5]. This suggests endothelium regulated islet survival.

Obesity induced insulin resistance is characterized by a compensatory increase in β-cell function and mass. Although the β-cell has the remarkable capacity to compensate for the higher insulin demand, this can eventually lead to β-cell overwork and a consequent loss of β-cell function and survival with manifestation of hyperglycemia and type 2 diabetes (T2D). Islet angiogenesis during compensated β-cell mass expansion and function has been highlighted in the ZDF rat, showing a transient islet hypervascularization preceding β-cell failure [6]. Other studies have revealed a dilation of pre-existing islet vessels in response to β-cell mass expansion [7] or an indispensability of proper vasculature during β-cell mass and function adaptation in response to high fat diet induced obesity in mice [8]. In contrast, a recent study shows that human islets present a higher vessel density in T2D, which occurs together with amyloid depositions in areas of thicker capillaries [9]. Such higher vessel area in T2D suggests that vessels, although they bring nutrients, which are especially important in the β-cell compensation state, may also be detrimental through the delivery of inflammatory products from the circulation to the proximity of the highly sensitive β-cells. Variation in the observations of the physiological relevance of islet vessel density on islet function may be due to differences in islet angio-architecture within and between species. Human islets were found to have lower vessel density than mouse islets [9] and whether islet capillaries favor proximity to β-cells is yet an evolving debate [10, 11].

Islet angiogenesis is promoted by three major families of angiogenic factors; vascular endothelial growth factors (VEGF), angiopoietins (Ang) and ephrins (Eph). Among the VEGFs, VEGF-A is most highly expressed in islets and most extensively studied in islet development and in the patho-physiology of diabetes. VEGF-A deficiency leads to a less dense and immature capillary network during embryonic [12, 13] as well as postnatal [14] islet development and reduces insulin content and secretion [15]. VEGF-A overexpression on the other hand, enhances islet vascularization but impairs islet morphogenesis and β-cell proliferation [16, 17]. The Ang/Tie family of angiogenic factors is so far poorly studied in the context of diabetes. It consists of the ligands Ang-1,-2 and Ang-4 (its mouse orthologue Ang-3) and the tyrosine kinase receptors Tie-1 and Tie-2. Ang-1 is expressed mainly by the perivascular cells and β-cells in mouse and human islets [14], and its agonist Tie-2 is expressed by the endothelial cells. Ang-2 expressed by endothelial cells classically antagonizes Tie-2 signaling [18]. It promotes vascular leakage [19] and is described as the pro-inflammatory angiogenic factor [20, 21], mostly studied in tumor vasculature. Ang-2 can also act as a context-dependent Tie-2 agonist [22, 23]. Thus, Ang-2 is known as a ‘multifaceted cytokine’ involved in angiogenesis as well as inflammation [24]. Adding to its ambiguity, Ang-2 has been considered as important part of the vascular niche involved in liver regeneration [25]. A dynamic regulation of Ang-2 by liver sinusoidal endothelial cells mediates hepatocyte proliferation by a complex cross-talk via TGFβ-1, thus highlighting its capacity in regenerative angiogenesis [26]. Tie-1 remains largely elusive in this system, though a co-operative role of Tie-1 in Ang-1/Tie-2 signaling ranging to a prominent role in vascular homeostasis has been indicated [27, 28].

Elevated levels of serum VEGF, Ang-2 and soluble Tie-2 have been associated with T2D and vascular dysfunction [29, 30]. Hyperglycemia and free fatty acids ablate Ang-1 mediated Tie-2 signaling in HUVECs [31] whereas Ang-1 protects islets from cytokine induced apoptosis also in absence of Tie-2 and improves islet revascularization post-transplantation [32]. In contrast, Ang-2 has been associated with vascular defects under hyperglycemia [33]. While VEGF-A overexpression increased vascularization near the islet cells and massively altered β-cell function, proliferation and mass, β-cell specific overexpression of Ang-1 or Ang-2 only slightly impaired insulin secretion and glucose tolerance together with marginal altered vascularization, islet mass and morphology [16]. The influence of diabetogenic conditions was not tested in these studies.

The question still remains, whether diabetes progression is a result from altered islet vascularization and whether an increased vascularization improves islet survival or rather promotes β-cell loss through inflammatory signals brought by the vessels. Therefore, in this study, we set to investigate the changes in islet vessels under diabetogenic conditions together with the function of Ang-2 in islet angiogenesis in the high fat/ high sucrose diet induced diabetes mouse model.

## Materials and Methods

### Animals

RIP-rtTA^tg/tg^ (C57BL/6/CBA background) and Tet-O-Ang-2^tg/wt^ (CD-1 background, backcrossed with C57BL/6) [16] mice were used to obtain β-cell specific Ang-2 overexpressing RIP-rtTA^tg/tg^; Tet-O-Ang-2 ^tg/wt^ mice (kindly provided by A. Powers, Nashville, TN). Briefly, we used the inducible “tet-on” (tetracycline regulated) transgenic system to allow expression of Ang-2 under the rat insulin promoter (RIP) by doxycycline (Dox; a tetracycline derivative). Male RIP-rtTA;Tet-O-Ang-2 and RIP-rtTA littermate control mice between 8 to 10 weeks of age were given a normal chow diet (ND) (Harlan Teklad Rodent Diet 8604 containing 12.2, 57.6, and 30.2% calories from fat, carbohydrate, and protein, respectively; Harlan Teklad, Madison, WI) or a high-fat high-sucrose diet (HFD) (“Surwit,” containing 58, 26, and 16% calories from fat, carbohydrate, and protein, respectively; Research Diets, Inc., New Brunswick, NJ) for 8, 16 or 24 weeks followed by islet isolation. The mice were provided with 1 mg/ml doxcycline in drinking water throughout the experiment with changes of water every 2 days. Male and female C57BL/6, 8–12 weeks of age were used for islet isolation and culture. All experiments were approved by the "Bremen Senate of health" (the Institutional Animal Care and Use Committee) in agreement with the §8 of the German animal protection law.

### Metabolic tests

Intraperitoneal glucose tolerance test (IPGTT): Mice were fasted for 14h overnight and injected intraperitoneally with 1g/kg body weight glucose (40%; B.Braun, Melsungen, Germany) and blood glucose levels were measured at 0, 15, 30, 60, 90, and 120 minutes post injection.

Intraperitoneal insulin tolerance test (IPITT): Mice were fasted for 4h and intraperitoneally injected with 0.75 IU/kg body weight of recombinant human insulin (InsHuman Rapid, Aventis, Germany) and blood glucose was measure 0, 15, 30, 60 and 90 minutes post injection.

Glucose-stimulated insulin release: Glucose stimulated insulin secretion was analysed in mice after 14h overnight fasting and intraperitoneal injection of 2 g/kg BW glucose. Blood samples obtained from the retrobulbar plexus at 0 and 30 minutes post injection and the serum was used to measure insulin concentration (Mouse ultrasensitive insulin ELISA, ALPCO Diagnostics, Salem, NH).

### Islet isolation and cell culture and treatment

Mouse islets were isolated as previously described [34]. Briefly, islets were isolated by 2 mg/ml liberase (Roche, Mannheim, Germany) injection into the pancreas and digested for 10 minutes at 37°C. Islets were purified by a density gradient of Histopaque (1:1; 1077 and 1119, Sigma) and by subsequent hand-picking. Human islets were isolated from six pancreases of healthy organ donors and from ten with T2D at the University of Illinois at Chicago, Lille University or at ProdoLabs. Informed consent was obtained from all subjects or their relatives and ethical approval given to the respective institutions. Research with human islets from brain dead donors applies to NIH regulations PHS 398, exemption 4. The use of human islets in the experiments have been approved by the "University of Bremen ethical committee". Islet purity was greater than 95% as judged by dithizone staining (if this degree of purity was not achieved by routine isolation, islets were handpicked). Mouse and human islets were cultured on extracellular matrix (ECM-) coated dishes (Novamed Ltd., Jerusalem, Israel, [35]) for 48h. For Ang-2 overexpression, islets from RIP-rtTA;Tet-O-Ang-2 and RIP-rtTA control mice were maintained in 10 μg/ml doxycycline prior to and during treatments. Islets were treated with diabetogenic conditions of glucolipotoxicity (22.2 mM glucose+ 0.5 mM palmitate) or a cytokine milieu (2 ng/ml IL-1β, 1000U/ml IFN-ɣ and TNF-α) for 3 days. MS-1 cells (mouse islet endothelial cell line ATCC CRL-2279) were cultured in DMEM 5.5 mM glucose/ 5% FCS and treated with glucolipotoxic (22.2 mM glucose+ 0.25 mM palmitate) or a cytokine milieu (2 ng/ml IL-1β, 1000U/ml IFN-ɣ and TNF-α) for 24h. Recombinant human Ang-2 (Peprotech) and 100 nM Tie-2 kinase inhibitor 4-(6-Methoxy-2-naphthyl)-2-(4-methylsulfinylphenyl)-5-(4-pyridyl)-1H-imidazole (CAS 948557-43-5; Calbiochem, Merck Millipore # 612085), a potent and selective Tie-2 tyrosine kinase inhibitor [36] was added in parallel to treatments. The highly selective Tie2 kinase inhibitor was developed by Semones *et al*. [36] by library screenings and in silico design and its in vitro and in vivo activity was shown [36, 37].

### Transfection and infection

Ang-2 or Tie-2 downregulation was achieved by using siRNA (ON-TARGETplus siRNA, Dharmacon) in dispersed islets. Transfection was carried out as previously described [38]. Briefly, islets were dispersed into smaller cell aggregates to increase transfection efficiency with accutase for 5–10 minutes at 37°C and then plated in ECM-coated dishes. 100 nM siScr or siRNA was delivered with Lipofectamine 2000 (Invitrogen). Ang-2 overexpression was obtained with control Adenovirus-GFP or Adenovirus-Ang-2 (kindly provided by H. Augustin, Heidelberg, Germany) 50MOI for 4h.

### Glucose stimulated insulin secretion of isolated islets

Mouse or human islets cultured in ECM dishes were stimulated by 2.8 mM glucose for 1h followed by 16.7 mM glucose for 1h at 37°C. Insulin content was extracted with 0.18 N HCl in 70% ethanol at 4°C overnight or RIPA lysis buffer (50mM Tris HCl pH 8, 150 mM NaCl, 1% NP-40, 0,5% Desoxycholate, 0,1% Sodium dodecyl sulfate, (SDS)) and determined by a human or mouse insulin ELISA kit (ALPCO).

### Immunocyto- and histo-chemical analysis

Isolated islets were fixed with 4% PFA/30 min. Mouse pancreases were isolated and fixed with 4% PFA for 8h at 4°C and then paraffin embedded and cut into 4μm sections. Human pancreas autopsies were obtained from the National Disease Research Interchange (NDRI). Autopsies were obtained from non-diabetic controls and from patients with poorly controlled diabetes, all with documented fasting plasma glucose >145 mg/dl (see S1 Table for age, gender and BMI). The slides were deparaffinized and immunostaining was carried out post heat antigen-retrieval. The dishes or slides were incubated with the primary antibodies; vessel marker- CD31 (ab28364, Abcam), proliferation- Ki67 (mouse-DAKO, human-Invitrogen), insulin (DAKO) or apoptosis- TUNEL-AP kit for dishes or TUNEL-Rx mixture for slides (Roche) followed by secondary anti-rabbit, -rat, -goat Cy3 or anti-guinea pig FITC or biotin conjugated antibodies (Jackson Immunosearch).

For morphometric analysis, ten sections (spanning the width of the pancreas) per mouse were analysed as described before [39]. Pancreatic tissue area and insulin-positive area were determined by computer-assisted measurements using a Nikon MEA53200 (Nikon GmbH, Dusseldorf, Germany) microscope and images were acquired using NIS-Elements software (Nikon). Mean percent β-cell fraction per pancreas was calculated as the ratio of insulin-positive and whole pancreatic tissue area. β-cell mass was obtained by multiplying the beta cell fraction by the weight of the pancreas. For quantification of blood vessels we measured islet and vessel area by a Region of interest (ROI) tool (vessel area) as well as by counting the number of vessels per islet (expressed as number of vessel per mm^2^; islet vessel number) using NIS-Elements software (Nikon) and ImageJ (v1.46), respectively.

### Gene expression analysis

Total RNA was isolated from islets or cells with a Trizol extraction system (TriFast-PEQLAB Biotechnology). For quantitative analysis of mRNA we used the Sybr Green real-time PCR kit (Applied biosystems). 18s rRNA or cyclophilin were used as internal housekeeping controls for all experiments and the products quantified by the ΔΔC_T_ method. Following primers were used: Mouse ANGPT1 Fw 5’-GCCACCATGCTTGAGATAGG-3’ and Rev 5’-TTCAAGTCGGGATGTTTGATT-3’, ANGPT2 Fw 5’-CGCTGGTGAAGAGTCCAACT-3’ and Rev 5’-ATTGTCCGAA-TCCTTTGTGC-3’, Tie-1/TIE Fw 5’-TCCCCCAGATCCTCAGTATG-3’ and Rev 5’-ATCTGGCTTGCGAAGTTTCA-3’, Tie-2/TEK Fw 5’-CCTTCACCAGGCTGATTGTT-3’ and Rev 5’-AATGCATTCCCCGGTATCTT-3’, CD31 Fw 5’-TGCTCTCGAA-GCCCAGTATT-3’ and Rev 5’-TGTGAATGTTGCTGGGTCAT-3’, NOS3 Fw 5’- GACCCTCACCGCTACAACAT-3’ and Rev 5’- CTGGCCTTCTGCTCATTTTC-3’, ICAM-1 Fw 5’-TGGCGGGAAAGTTCCTGTTT-3’ and Rev 5’-TAGGAGATGGG-TTCCCCCAG-3’.Human ANGPT1 Fw 5’-ATCCCTCCGGTGAATATTGG-3’ and Rev 5’-GAATAGGCTCGGTTCCCTTC-3’, ANGPT2 Fw 5’-GGCTGGGAAATGAGTT-TGTT-3’ and Rev 5’-CGGCTGTCCCTGTAAGTCCT-3’, Tie-1/TIE Fw 5’-GACTCC-GAGATCCAGCTGAC-3’ and Rev 5’-CCTGTCCACGTCTATCCACA-3’, Tie-2/TEK Fw 5’-TGCTGTCATCAACATCAGCTC-3’ and Rev 5’-TGTTGCCAAGCCTCAT-AGTG-3’, CD31 Fw 5’-ATGATGCCCAGTTTGAGGTC-3’ and Rev 5’-ACGTC-TTCAGTGGGGTTGTC-3’, NOS3 Fw 5’- ACCCTCACCGCTACAACATC -3’ and Rev 5’- GCTCATTCTCCAGGTGCTTC -3’, ICAM-1 Fw 5’-AACTGGACGTGGCC-AGAAAA -3’ and Rev 5’- ACAGAGGTAGGTGCCCTCAA -3’.

### Western blot analysis

Islet and cell protein was extracted using RIPA lysis buffer and 25 ug protein was loaded for western blot analysis. Actin, tubulin, cleaved caspase 3, myc (Cell Signalling Technology, Inc.), phospho-Tie-2 (Y992, R&D systems), Ang-2 and ICAM-1 (F-18 and G-5, Santa Cruz BioTechnology, Inc.) were used at a 1:1000 dilution.

### Statistical analysis

All values are expressed as the means ± SEM. The different groups were compared by Students t-test and ANOVA with a Bonferroni post-hoc analysis. One-way Anova was used for all analyses except two-way ANOVA for 2 variables was used in mouse in vivo studies (changes in diet and Ang2-expression). P value<0.05 was considered statistically significant.

## Results

### Islet vessel area increases in T2D

**Fig 1:**
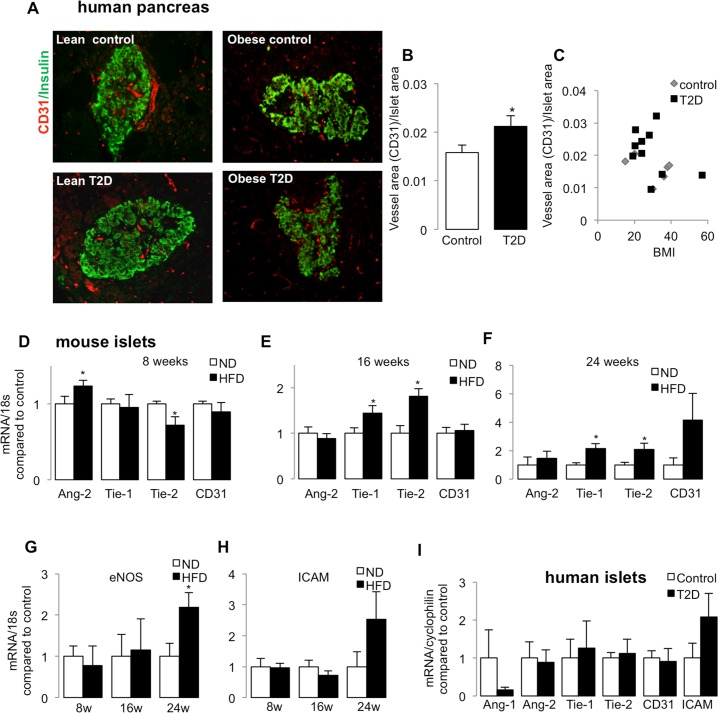
Islet vessel area increases in T2DM. **(A)** Representative images of pancreatic sections from non-diabetic controls and patients with T2D, immune-labelled for CD31 (red) and insulin (green). **(B)** Graphs show ratio of vessel area to islet area (control: n = 6; T2D: n = 10). **(C)** Plot shows no correlation of vessel density with BMI. **(D-H)** qPCR analysis of Ang-2, Tie-1, Tie-2, CD-31 from isolated mouse islets from C57BL/6 WT mice kept on normal diet (ND) or high-fat high-sucrose diet (HFD) for **(D)** 8 weeks (n = 4/group), **(E)** 16 weeks (n = 9/group) and **(F)** 24 weeks (n = 7/group), **(G) of** eNOS and **(H) of** ICAM-1. **(I)** qPCR analysis of Ang-1, Ang-2, Tie-1 and Tie-2 of isolated islets from non-diabetic (control, n = 8) and from patients with T2D (n = 7). *p<0.05, HFD vs ND or T2D vs. control

A causative correlation of islet vessel density and diabetes progression has been suggested for years but it was not clearly known whether there are increased or reduced vessels in islets in diabetes. Using autopsy pancreases, we quantified vessel area within the pancreatic islets by CD31/PECAM-1, located on the surface of endothelial cells and various blood cells. In full accordance with the recent study by Brissova *et al*. [9], there was a 1.3-fold increase in vessel area to islet area in T2D donors (n = 10) compared to non-diabetic controls (n = 6; Fig 1A and 1B; the donor information is provided in S1 Table). Both lean and obese individuals were included in the analyses. We found no correlation in islet vessel area and BMI, neither in non-diabetic nor in diabetic donors (Fig 1C), suggesting that vessel area, then normalized to islet area, was not affected by the BMI.

We further investigated the molecular basis of such changes in vessels in a model of diabetes progression in mice fed a high-fat high-sucrose diet (HFD; Surwit [40]) up to 24 weeks. Increased islet vessel area but reduced intraislet vessel density was shown in several mouse models of obesity and insulin resistance, including HFD feeding [7]. Angiogenic profile analysis after 8 weeks of HFD feeding, which already affects glucose tolerance [41] showed upregulation in Ang-2 (1.2-fold) and reduction of its receptor Tie-2 on endothelial cells compared to normal diet (ND) fed mice but other vessel markers (Tie-1, CD31) remained unchanged (Fig 1D). Ang-1 is constitutively expressed at low levels, mainly by pericytes. In mouse islets, its expression was rarely detectable and was unchanged (8 weeks) and further downregulated with time of HFD feeding (data not shown), the same trend of reduction also occurred in human islets isolated from patients with T2D (Fig 1I, p = 0.08). At 16 weeks of HFD, when robust glucose intolerance occurs together with β-cell apoptosis [39, 42], both angiopoietin receptors Tie-1 and Tie-2 were upregulated (1.5- and 1.8-fold, compared to ND control, Fig 1E), which remained higher after 24 weeks HFD (Fig 1F). This was paralleled by an increase in eNOS, which is involved in vessel dilation (2.2-fold, Fig 1G), and a strong tendency of ICAM-1 increase (2.5-fold, Fig 1H) in HFD as well as in human T2D islets (Fig 1I), a marker known to be associated with leukocyte infiltration. No significant change in islet insulin mRNA was seen in HFD mice compared to ND (S1A–S1C Fig). VEGF-A upregulation was more prominent at 16 weeks (S1D Fig), as shown previously [17]. Thus, the angiogenic profile adapts with an increase in the angiopoietin receptors Tie-1 and Tie-2 during vessel expansion and hypervascularization [6] during the progression to T2D.

### Ang/Tie expression in isolated islets correlates with changes in vessel area

**Fig 2:**
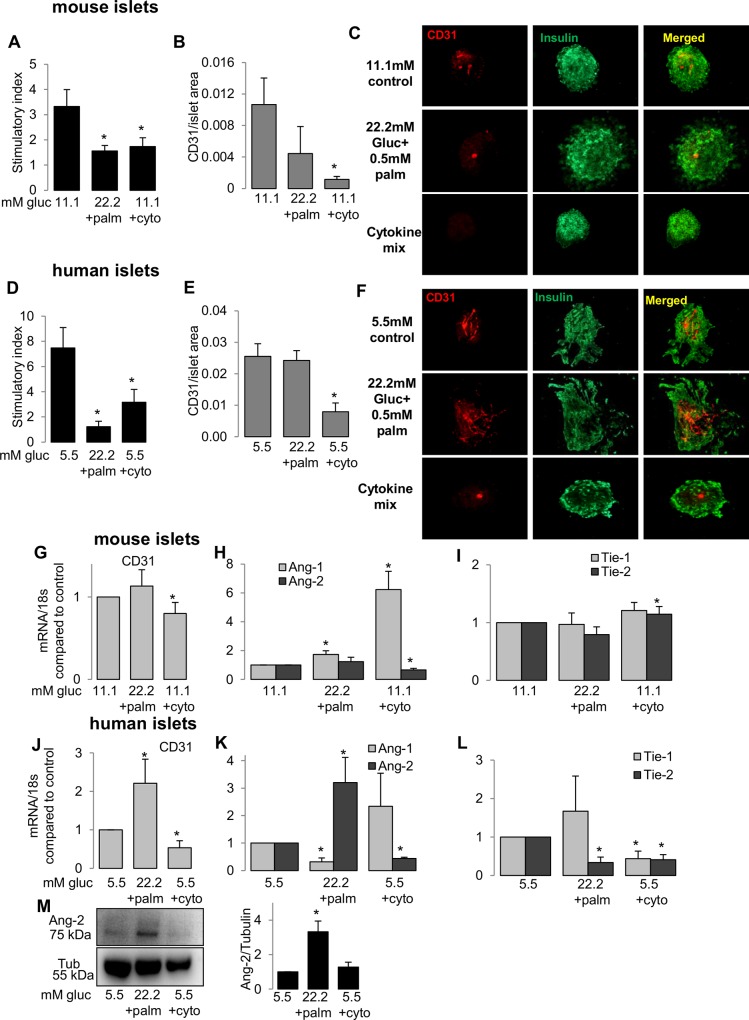
Ang/Tie expression in isolated islets correlates with changes in vessel area. Isolated WT mouse and human islets were cultured for 3 days in control condition (11.1 mM glucose for mouse or 5.5 mM for human) or treated with diabetic conditions of 22.2 mM glucose + 0.5 mM palmitic acid or mixture of cytokines: 2 ng/mL IL-1β, 1000 U/ml IFN-ɣ and TNF-α (cyto). **(A,D)** GSIS is shown by the stimulatory index assessed by 16.7/2.8 mM glucose stimulation. **(B,C,E,F)** Graph shows ratio of vessel area to islet area for mouse **(B,C)** and human **(E,F)** islets, fixed and immune-labelled for vessel (CD-31,red) and islet (insulin, green). **(G-L)** qPCR analysis of treated mouse and human islets for mouse CD-31 **(G,J)**, Ang-1,-2 **(H,K)**, Tie-1,-2 **(I,L)**. All genes have been normalized to PPIA or 18s as housekeeping control. *p<0.05, treated vs. control 11.1 mM (mouse) or 5.5 mM (human). **(M)** Representative western blot from treated human islet lysates (left panel) and densitometric analyses of Ang-2 (right panel). Data are means +/-SE from 3–5 independent experiments from 3–5 different organ donors (human islets) or 3–5 independent mouse islet isolations.

We further asked the question whether such increase in Tie-1/2 receptors is relevant for β-cell failure in T2D progression. Isolated mouse and human islets were used to study the interactions between islet endothelial cells and islet function. To maintain their physiological integrity, mouse and human isolated islets were cultured on extracellular matrix coated dishes [43] and treated with a diabetic milieu of high glucose (22.2 mM) and palmitic acid (0.5 mM) to mimic glucolipotoxicity or with a mixture of cytokines (cyto; IL1-β, IFN-ɣ and TNF-α). Mouse as well as human islets showed impaired insulin secretion in response to glucose under glucolipotoxicity and cytokines (Fig 2A and 2D).Though β-cell function was impaired in both conditions, we found a clear reduction in the endothelial cell area only under cytokine- and not under gluco-lipotoxic conditions in both mouse (90% reduction) and human (72% reduction) islets (Fig 2B and 2E) by double immuno-labelling for CD31 (endothelial cells) and insulin (β-cells; Fig 2C and 2F). The persistent vessels under elevated glucose may support the higher vessel density observation in T2D, while cytokine treatment seems to be deleterious for both β- and endothelial cells.

In sync to the differences in vessel area under both diabetic conditions we also saw an opposing trend in expression of Angiopoietin/Tie angiogenic factors and CD31 mRNA in islets.

CD31 expression was downregulated under cytokine treatment in both mouse (20% reduction) and human islets (47% reduction; Fig 2G and 2J). While Ang-1, the agonist for Tie-2, was upregulated under cytokine treatment in mouse islets (6.2-fold) and also with a tendency in human islets, Ang-2, the classical antagonist was oppositely lower under cytokines in both mouse (34% reduction) and human islets (56% reduction; Fig 2H, 2K and 2M). Both, Tie-1 and -2 expression was also down-regulated under cytokines in human islets (Fig 2L), with unchanged Tie-1 and rather increased Tie-2 in mouse islets.

The diabetogenic condition of glucose/palmitate rather reflected a state of compensating by angiogenic factors; with upregulated CD31 expression in human islets (2.2-fold; Fig 2G) and oppositely to the cytokine treatment lower Ang-1 and higher Ang-2 (3.2-fold; Fig 2K and 2M) in human islets, while there was almost no change in expression in mouse islets.

### Ang-2 over-expression basally impairs islet function but protects from cytokine induced apoptosis in isolated islets

Seeing the upregulation of Ang-2 by glucolipotoxicity and its reduction by cytokines in human islets, we were intrigued by the ambiguous role of Ang-2 and its direct or indirect- via endothelial cells, effect on islet function in diabetes.

We initially used a downregulation approach to model the situation under cytokines to see if there is an effect on islet survival or function. Downregulation of Ang-2 using siRNA in human islets had neither an effect on glucose stimulated insulin secretion nor on islet survival, although ICAM was upregulated by Ang-2 silencing in the cytokine condition (S2A and S2B Fig).

**Fig 3:**
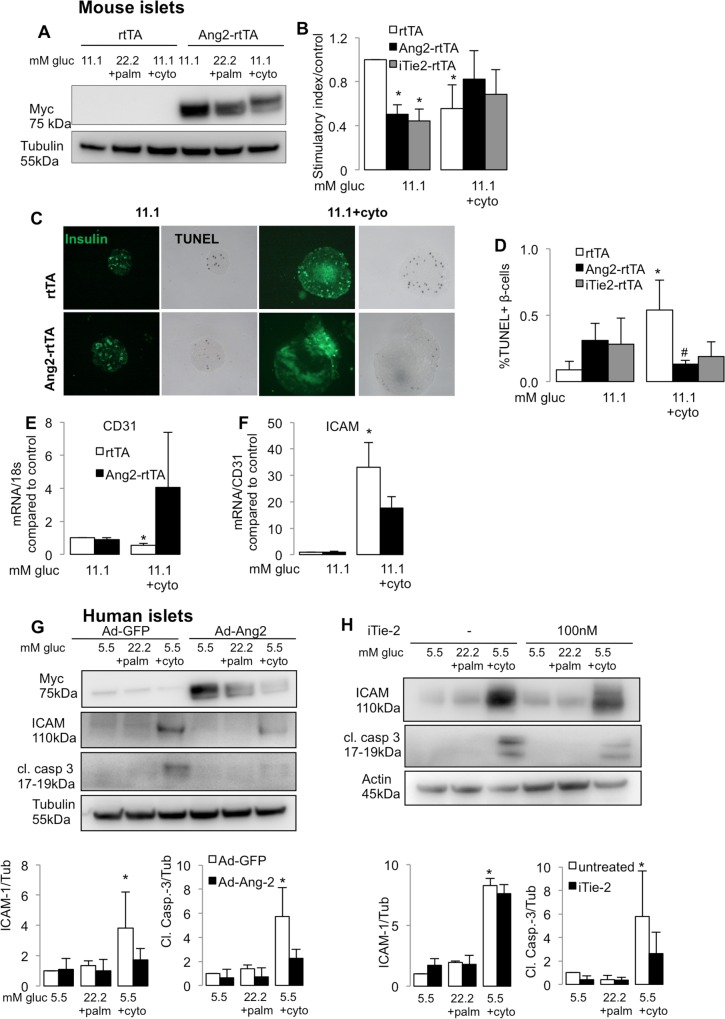
Ang-2 over-expression impairs islet function but protects from cytokine treatment in isolated islets. Isolated islets from RIP-rtTA;tet-O-Ang-2 (Ang2-rtTA) and RIP-rtTA control (rtTA) mice were cultured for 3 days in presence of 10 μg/ml doxycycline to achieve Ang-2 overexpression. Mouse or human islets were cultured in 11.1 (mouse) or 5.5 mM glucose (human) or treated with diabetic conditions of 22.2 mM glucose + 0.5mM palmitic acid or mixture of cytokines: 2 ng/mL IL-1β, 1000 U/ml IFN-ɣ and TNF-α (cyto). **(A)** Western blot from treated mouse islets shows Ang-2 overexpression in islets by myc-Ang-2. **(B)** GSIS is shown by the stimulatory index assessed by 16.7/2.8 mM glucose stimulation and normalized to control. **(C,D)** Treated mouse islets fixed post-GSIS and apoptotic cells detected by double staining for TUNEL and insulin. Representative images from different treatments. **(E,F)** qPCR analysis for CD31 **(E)** and ICAM **(F)** from mouse islets overexpressing Ang-2. **(G,H)** Representative western blots (upper panel) and densitometric analyses of proteins (lower panels) showing myc-Ang-2, ICAM-1, cleaved caspase 3 and actin/tubulin as housekeeping control, in human islets overexpressing Ang-2 by Ad-Ang-2 or control Ad-GFP **(G;** MOI = 50) or treated with 100 nM Tie-2 inhibitor for 72h (**H)**. Data are means +/-SE from 3–5 independent experiments from 3–5 different organ donors (human islets) or 3–5 independent mouse islet isolations. *p<0.05, treated vs. 11.1 mM glucose control, #p<0.05, Ang2-rtTA vs. rtTA.

Ang-2 was then overexpressed to investigate whether antagonistic Tie2 signals promote β-cell function and survival. β-cell specific Ang-2 upregulation was obtained in islets isolated from mice expressing myc-tagged Ang-2 under the rat insulin promoter conditionally via the reverse tetracycline activator (RIP-rtTA;Tet-O-Ang-2)[16] and their culture in presence of doxycycline (Fig 3A). Ang-2 over-expression remarkably impaired islet function at basal conditions (50% reduction) as indicated by the GSIS. In contrast, Ang-2 overexpression had no significant effect in cytokine treated mouse islets, where GSIS was impaired, (Fig 3B).

Ang-2 not only has a dual role on Tie-2 signaling with antagonistic as well as agonistic effects, but also interacts with other receptors like the integrins [21, 22]. A Tie-2 kinase inhibitor fully modulated the effects of Ang-2 overexpression with reduction in β-cell function at basal level and no effect under cytokine treatment, suggesting that the Ang-2 effects may be Tie-2 specific (Fig 3B).

We also investigated the effect of Ang2/Tie2 on β-cell survival by analyzing apoptosis by the TUNEL-assay (Fig 3C and 3D) and proliferation by Ki-67 staining (S2I Fig) in mouse islets. No effect on proliferation but protection from cytokine-induced apoptosis was seen with Ang-2 over-expression in mouse islets (41% reduction, rtTA vs. Ang2-rtTA; Fig 3D). Western blot analyses from treated human islet lysates revealed a similar protection from cytokine induced apoptosis with Ang-2 overexpression and Tie-2 inhibition, seen by lower cleaved caspase 3 (Fig 3G and 3H).

Along with effects on survival, Ang-2 overexpression and Tie-2 inhibition reduced the expression of endothelial inflammatory marker ICAM-1 in mouse (Fig 3F) and human islets (Fig 3G and 3H). The effect of Tie-2 inhibition was reproducible by downregulation of Tie-2 using siRNA in human islets (S2B Fig) and in the mouse endothelial cell line MS-1 (S2C and S2D Fig); Tie-2 inhibition also resulted in reduced caspase 3 in endothelial cells under cytotoxic conditions (S2C and S2D Fig). Efficacy of the Tie-2 inhibitor to reduce pTie-2 was shown in a proof-of-principle experiment. Oppositely, Ang-2 increased pTie-2 in the absence of Ang-1 in MS-1 cells (S2E Fig). Ang-2 and Tie-2 were successfully downregulated by siRNA (S2F–S2H Fig).

We could confirm the protective role of Ang-2 at cytotoxic conditions by the opposite experiments. Downregulation of Ang-2 exacerbated inflammation, seen by tendency of ICAM-1 upregulation in MS-1 cells (S2J Fig) and human islets under cytotoxic conditions (S2B Fig), but had no additional effect on GSIS or β-cell survival, neither at control nor at diabetogenic conditions.

Thus, inhibition of Tie-2 signaling at basal conditions impairs insulin secretion. In contrast, when vessels are lost and Ang-2 expression is pathologically reduced by cytokine treatment, its overexpression protects from cytokine-induced apoptosis. This indicates towards a classic antagonistic role of Ang-2 on Tie-2 signaling.

### Ang-2 over-expression leads to islet hypovascularization and β-cell failure in response to HFD

**Fig 4:**
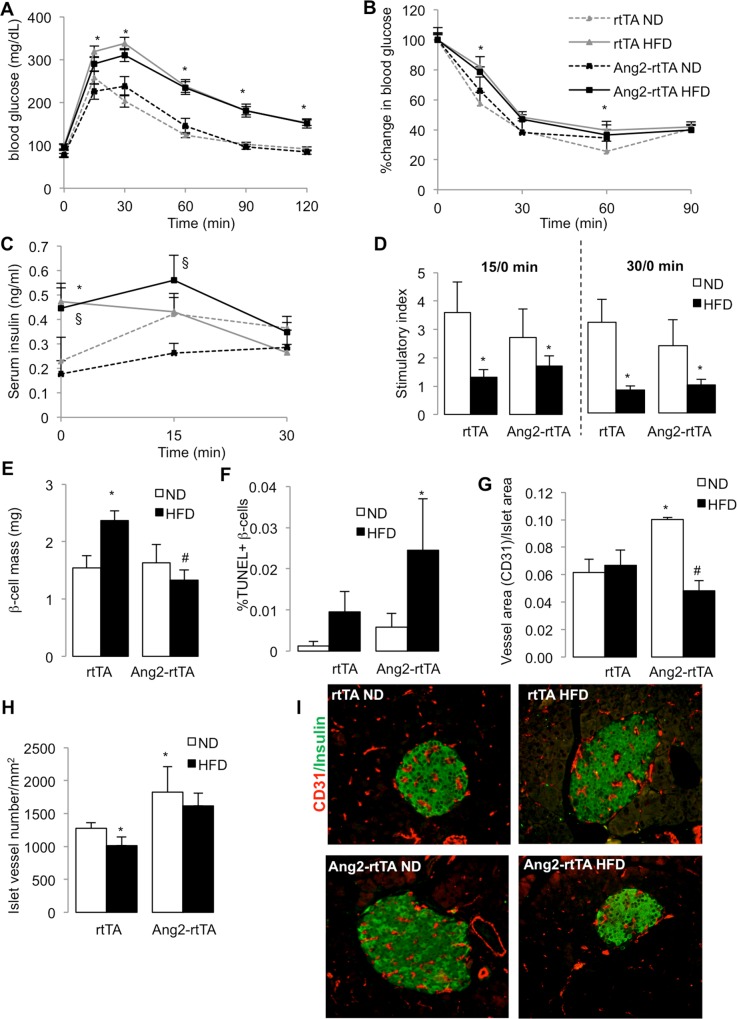
Ang-2 over-expression leads to islet hypovascularization and β-cell failure in response to HFD. β-cell specific overexpressing male Rip-rtTA;tet-O-Ang-2 (Ang2-rtTA) and Rip-rtTA (rtTA) kept on a normal diet (ND) or high-fat high-sucrose diet (HFD) received 1mg/ml doxycycline in drinking water for 16 weeks. **(A,B)** Blood glucose levels from intraperitoneal glucose **(A)** and insulin tolerance **(B)** tests performed at 16 weeks of HFD. **(C,D)** Glucose stimulated insulin secretion, showing levels of serum insulin and stimulatory index at 15 and 30 min after glucose injection. Data are means +/-SE from 4 independent experiments from n = 8 (ND) or n = 19 mice/group. **(E-H)** Pancreatic sections were analysed for **(E)** β-cell mass **(F)** apoptosis by double staining for TUNEL and insulin (10,000 β-cells/mouse, n = 6 mice/group) and **(G)** islet vessel area quantified by CD31/Insulin co-staining (100 islets/mouse, n = 6 mice/group). **(H)** Islet vessel density from 3 animals per group represented as number of vessels per islet-mm^2^ **(I)** Representative images from CD31 (red)/Insulin (green) co-staining. *p<0.05 vs. rtTA ND, ^#^p<0.05 vs. rtTA HFD, ^§^p<0.05 vs. Ang2-rtTA ND

Ang-2 was transiently upregulated under HFD and islets were hypervascularized towards progression to T2D from 8 weeks to 24 weeks in mice, as well as under glucolipotoxicity *in vitro*. Thus we set to investigate whether Ang-2 plays a role in causing hypervascularization. Rip-rtTA;tet-O-Ang-2 and control Rip-rtTA [16] littermates were fed a high-fat high-sucrose diet and Ang-2 expression was induced by doxycycline in the drinking water for 16 weeks during the diet. β-cell-Ang-2 overexpression was confirmed in the Rip-rtTA;tet-O-Ang-2 mice (S3A Fig). During the 16 weeks feeding, HFD fed mice gained 46% and ND fed mice gained 9.3% weight; there was no effect on weight gain or food intake by Ang-2 overexpression (S3B and S3C Fig). HFD feeding lead to impaired glucose and insulin tolerance, compared to ND fed mice. Ang-2 overexpression did not have any significant effect on glucose homeostasis under ND or HFD (Fig 4A and 4B). HFD-feeding resulted in markedly higher fasted serum insulin compared to the ND fed mice, sharp attenuation of insulin secretion at 15 min post i.p. glucose challenge (Fig 4C, p<0.05) and fully abolished glucose stimulated insulin secretion (Fig 4D, p<0.05).

In line with a previous study [16], Ang-2 had a tendency for impairment in insulin secretion under normal diet (p = 0.06 vs. ND control), but the Ang-2-HFD mice showed significantly higher insulin at 15 min post glucose injection. This did not reflect in changes of the stimulatory indices when compared to control ND or HFD mice (Fig 4D). Therefore, it was confirmed that post-natal Ang-2 overexpression over 4 months in the β-cells induces a minor impairment in insulin secretion under normal diet but it does not affect glucose homeostasis in response to HFD.

Ang-2 overexpression also did not change glycemia and insulin secretion in the MLD-STZ-mouse model (after 5 consecutive injections of 50 mg/kg streptozotocin; data not shown), which reflected the neutral effects of Ang-2 overexpression on GSIS upon cytokine treatment in isolated mouse islets.

In WT mice HFD feeding resulted in a compensatory increase in β-cell mass, compared to ND fed mice (Fig 4E). Ang-2 over-expression had no effect at ND, but resulted in a lower β-cell mass in the HFD (Fig 4E).

To address whether this reduction in β-cell mass might be stemming from an effect on β-cell survival, we looked at proliferation and apoptosis, using Ki-67 and TUNEL-assay, respectively. There was no significant difference in cell proliferation with diet or Ang-2 expression (S3D Fig) but increased β-cell apoptosis by Ang-2 overexpression (Fig 4F).

There was no change in the proportion of the islet vessel area to islet area under the HFD after 16 weeks, but a significant reduction in islet vessel density (Fig 4G and 4H). Under ND, Ang-2 overexpression significantly increased the islet vessel area and number (Fig 4G and 4H), but together with increased β-cell apoptosis, a significant reduction in islet area together with an only marginal and insignificant reduction in density occurred by Ang-2 under the HFD. The vascular changes induced by Ang-2 overexpression were also reflected by Ang-1 mRNA downregulation (S3E Fig) and upregulation of VEGF-A, Tie-1, Tie-2 and CD31 (S3F Fig) under ND. Thus, under physiological conditions Ang-2 induces islet vessel area as well as density, while under HFD, islet vessel area is reduced. HFD-induced endothelial inflammatory status worsened with Ang-2 overexpression as seen by induction VCAM-1 (S3H Fig) and E-Selectin (S3I Fig), involved in leukocyte infiltration, which may directly lead to increased β-cell apoptosis, although a further functional decline did not occur.

## Discussion

In this study we report an increased islet vessel area in T2D, which is in agreement with a recent study [9]. Such observation was rather surprising, since it is known for several years, that either a lower islet vessel density or a dysfunctional endothelium is paralleled with diabetes progression, although such has never been shown in the human pancreas. When normalized to islet area, increased vessels were observed in T2D, but no change in vessel density was observed with increased BMI. This suggests, that vessel growth compensation is paralleled with β-cell mass adaptation in obesity in the human pancreas as well as during high fat feeding in the mouse pancreas.

The increased vessel density in human T2D pancreata was based on the induction in CD31 protein. Such CD31 increase could not be confirmed in isolated T2D islets on the mRNA level, although CD31 staining was clearly seen in islets after isolation. It is possible, that such mRNA changes cannot be seen after the harsh process of islet isolation and that subsequent 24h culture under the same normoglycemic conditions may result in a loss of such changes between non diabetic and diabetic islets. Similarly, we also did not observe changes in Tie-1/Tie-2 mRNA levels in the human T2D islets, as seen in mouse islets under prolonged hyperglycemia induced by high fat/ high sucrose feeding.

The importance of a physiological balance of islet vascularization by adaptive angiogenesis for β-cell mass expansion and function was questioned; shown to be negligible on one [7, 8], but to be indispensible on the other hand [44]. Especially the angiogenic factor VEGF-A promotes islet re-vascularization but directly impairs β-cell proliferation leading to a progressive loss in β-cell mass in response to HFD [14, 17]. Also enhanced c-Kit receptor signaling improves islet vasculature and function in aged mice but elicits an inflammatory response and impairs islet function in response to HFD [45]. VEGF-A [14, 15, 46] and several endothelial derived molecules, collagen IV, thrombospondin-1 [47] and β1-laminin modulate islet function *in vitro*. Endothelial- β-cell co-culture leads to the formation of pseudoislets [48], and endothelial conditioned medium improves islet function [49]. Ang-1/Tie-2 signaling promotes cell-cell contacts and contact to extracellular matrix [50, 51].

**Fig 5:**
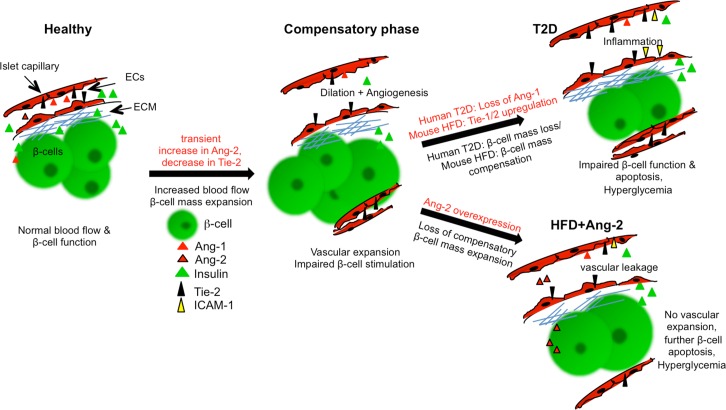
Islet hypervascularization in T2D and effects of Ang-2 overexpression. A healthy islet is surrounded by intact capillaries, maintained by the Ang/Tie system and extracellular matrix supporting the function and survival of the islet. An increased insulin demand leads to more islet blood flow, β-cell mass and vascular expansion and consequent compensation. A transient Ang-2 upregulation promotes angiogenesis, leading islet endothelium to a non-quiescent state inhibiting Tie-2 signaling. Towards human T2D progression, β-cell failure and apoptosis occurs together with increased islet and endothelial inflammation and islet hypervascularization. Ang-2 overexpression on the other hand prevents β-cell mass and vascular expansion in response to HFD with persistent islet and endothelial inflammation.

The Ang/Tie angiogenic cascade also participated in the adaptive increase in islet vascularization with Ang-2 upregulation early during high fat/ high sucrose feeding in mice (compensatory phase- cartoon Fig 5) and a persistent Tie receptor upregulation through 24 weeks of the diabetogenic diet, together with upregulated vessel markers eNOS and CD31 at 24 weeks on a high fat diet and ICAM-1 also in human T2D islets.

Ang-2 induced vascular defects have been observed under hyperglycemia in chick pancreases transplanted to STZ-treated mice [33]. This implies a role of Ang-2 in inducing vascular anomalies. A resultant compensatory upregulation of Tie-1/-2 to restore vessel stabilization [52] might be in play as seen at 24 weeks of HFD. Ang/Tie were differentially regulated during HFD feeding and we hypothesized a role for Tie signaling driving islet angiogenesis and function in T2D.

HFD induced reduction in islet vessel number is in line with previous observation which also show reduced islet vessel density [7]. Ang-2 overexpression caused islet hypervascularization with a slight impairment in insulin secretion under a normal diet, owing to the vascular defects causing irregular blood perfusion (Ang-2+HFD–scheme Fig 5). Despite this minor defect, there was no change in glucose tolerance or insulin sensitivity in Ang-2 overexpressing mice.

On the other hand, in the HFD-induced obesity and insulin resistance model, β-cell specific Ang-2 overexpression led to a reduced vessel area with a minor decrease in vessel number, reduced β-cell mass with increased β-cell apoptosis. This indicates Ang-2 induced endothelial apoptosis leading to vessel fragmentation shown by a reduced vessel area but not number. From this data, one can clearly assume, that adaptive hypervascularization is indeed necessary for the maintenance of β-cell survival under situations of higher insulin demand by the β-cell.

Hypervascularization in T2D was supported by our findings in human autopsies and mouse islets from HFD fed mice as well as in vitro studies in mouse and human islets. Significantly upregulated levels of Ang-2 under glucolipotoxicity occurred along with a persistent vessel area despite complete ablation of β-cell function.

Impairment in Tie-2 receptor signaling in hyperglycemia and elevated fatty acids via Ang-1 downregulation has been reported [31]. Thus, the inverse regulation of Ang-1/Tie-2 and Ang-2 in glucolipotoxicity points to a rather beneficial role of Tie-2 signaling in islets. Glucolipotoxicity in β-cell failure is classically known [53, 54] but the endothelial cell number is reduced in parallel to β-cell dysfunction in the culture conditions we used.

While glucolipotoxicity induced CD31 expression together with an increase in Ang-2 in human islets, pro-inflammatory cytokines had a drastic effect on the endothelial cells; with loss in CD31 and Ang-2 downregulation. We did not observe any effect on β-cell survival upon modulating Ang-2 or Tie-2 in cultured islets under glucolipotoxicity. But at the conditions of reduced islet vessel density reduced Ang-2 at cytokine treatment *in vitro*, Ang-2 overexpression significantly prevented cytokine-induced apoptosis in mouse and human islets, pointing again to the importance of maintaining physiological balance of angiogenic factors.

The protective effect of Ang-2 on cytokine induced apoptosis in islets and re-vascularization has already been shown [32]. This protection was also induced by Ang-2, rather classically known to be pro-inflammatory, e.g. by sensitizing endothelial cells to the effect of TNFα [21], but also shown to be anti-apoptotic in lymphatic endothelial cells [55]. Ang-2 can assert beneficial effects in a Tie-2 independent manner through integrins [22]. In this study, Ang-2 triggered β-cell apoptosis in HFD-induced diabetes, confirming its widely studied role in vascular leakage [19, 20]. This was supported by the VCAM-1 and E-selectin upregulation in islets from Ang-2-overexpressing HFD mice. In contrast, we also show the opposing Ang-2 beneficial effects *in vitro* on apoptosis protection, which were confirmed by inhibiting Tie-2 *in vitro* in human islets and in the mouse endothelial cell line. In contrast to its protective role on apoptosis, in vitro β-cell function under basal conditions was impaired by Ang-2 as well as by Tie-2 inhibition, affirming the antagonistic role of Ang-2 in pathophysiological conditions. Our study suggests, that only under situations of lost vessels, as seen under cytokine treatment, additional Ang-2 to balance its action is protective. Further reduction of Ang-2, even under conditions of lost vessels had no potentiating effect on function and death, but the downregulation of Tie-2, the opposite player of Ang-2 signals, had a protective effect on survival, similar to the Ang2 upregulation.

## Conclusions

Although Ang2 exerts various effects in isolated human and rodent islets; under diabetogenic conditions as well as in *in vitro* and *in vivo*, it becomes clear that an imbalance in angiogenic factors is deleterious for homeostasis. This may also explain its ambiguous effects. Our study shows that a functional vascular adaptation together with the physiological equilibrium of Ang-2/ Tie-2 signaling under diabetic conditions is highly important for maintaining β-cell survival and function.

## Supporting Information

S1 Fig qPCR analysis of isolated mouse islets from C57BL/6 WT mice kept on normal diet (ND) or high-fat high-sucrose diet (HFD) for insulin genes Ins1, Ins2 at **(A)** 8 weeks, **(B)** 16 weeks **(C)** 24 weeks, **(D)** VEGF-A expression at 8, 16 and 24 weeks. All genes were normalized to PPIA. Data are means +/-SE from 3–4 mice/group from independent mouse islet isolations.
(TIF)

S2 Fig **(A,B)** Human islets were transfected with siRNA (siAng-2 or siTie-2) and control siScr. Islets were treated with diabetic conditions of 22.2 mM glucose + 0.5 mM palmitic acid or mixture of cytokines 2 ng/mL IL-1β, 1000U IFN-ɣ and TNF-α (cyto) for 72h. **(A)** GSIS is shown by the stimulatory index assessed by 16.7/2.8 mM glucose stimulation and normalized to control. **(B)** Western blot analyses of human islet lysates showing ICAM-1, cleaved caspase-3 and actin. **(C-H)** Western blot analyses **(C-E,G)** or RT-PCR **(F,H)** of MS-1 cells, transfected with siRNA (siTie-2. siAng-2) and control siScr or cultured with recombinant Ang-2 or Tie-2 inhibitor and treated as above for 24h. Blots show Ang-2, ICAM-1, cleaved caspase-3 and actin **(C,D)** and pTie-2, CD31 and Actin **(E)**. **(F-H)** Data showing RNA **(F,H)** and protein **(G)** downregulation. (**I**) Isolated islets from RIP-rtTA;tet-O-Ang-2 and RIP-rtTA mice were cultured for 72h in presence of 10 μg/ml doxycycline for Ang-2 overexpression and treated as above, fixed post-GSIS and co-labelled with Ki-67/Insulin, Graph showing %Ki-67 positive β-cells. **(J)** qPCR data for Ang-2, CD31 and ICAM-1 in MS-1 cells transfected with siAng-2 or control siScr and treated as above for 24h. Data show means +/-SE from 3–4 independent experiments from 3 different organ donors (human islets) or 3 independent mouse islet isolations (A,B,F-J). C-E are single experiments *p<0.05 vs. Ang-2-rtTA or siScr 5.5
(TIF)

S3 Fig β-cell specific overexpressing male Rip-rtTA;tet-O-Ang2 and Rip-rtTA were kept on a normal diet or high-fat high-sucrose diet for 16 weeks. **(A)** Representative images from mouse pancreatic sections showing β-cell specific Ang-2 expression (red) in mouse β-cells (insulin; green). **(B)** % weight gain and **(C)** food intake/mouse/day over 16 weeks. Data are means +/-SE from 4 independent experiments from n = 8 (ND) or n = 19 mice/group. *p<0.05 vs rtTA ND. **(D)** %Ki-67 positive β-cells in mouse pancreatic sections, n = 6 mice/group **(E-I)** qPCR analyses from 1 single experiment from pooled isolated islets from 4 mice per treatment group for Ang-2, Ang-1, VEGF-A, Tie-1, -2, CD31, ICAM-1, eNOS, VCAM-1, E-Selectin of islets isolated from rtTA and Ang-2-rtTA, ND and HFD mice at 16 weeks. Because of the cell pooling strategy before analysis, no statistical analysis could be performed.
(TIF)

S1 Table Donor information–age, gender and BMI, of autopsies obtained from non-diabetic controls and patients with T2D. (TIF)
