# Correction: Angiopoetin-2 Signals Do Not Mediate the Hypervascularization of Islets in Type 2 Diabetes

**DOI:** 10.1371/journal.pone.0282771

**Published:** 2023-03-02

**Authors:** Payal Shah, Navina Lueschen, Amin Ardestani, Jose Oberholzer, Johan Olerud, Per-Ola Carlsson, Kathrin Maedler

After this article [1] was published, concerns were raised about some of the statistical analyses in this article. *PLOS ONE* evaluated the concerns in consultation with an Academic Editor and statistical reviewer, who advised that:

- Parametric tests, including t-tests and ANOVAs, were not appropriate given the small sample size of 10 vs. 10 in article [1].
- The study was not sufficiently powered to reject the null hypothesis, and this was not discussed as a study limitation.

The corresponding author disagreed with the concern about the use of parametric statistical tests in the study, but provided non-parametric Wilcoxon Signed Rank Test analyses for the experiments in Figs 1B, 2B, 2D-E, 3B, 4A-B and 4D-G (S3 File).

- Results reported in Figs 1B, 2B, 2D, 2E, 4E, 4F, 4G, and the comparison of rtTA to Ang2-rtTA and iTie2-rtTA in Fig 3B, are significant by both t-test and Wilcoxon test, and therefore these results and the corresponding conclusions are unchanged.
- Wilcoxon test results for the Fig 3B comparison of rtTA to rtTA+cyto indicated no significant difference in means between rtTA and rtTA+cyto (p = 0.31), contrary to results obtained by t-test.
- The Fig 4D results are consistent when comparing results of the t-test to the Wilcoxon test, except for comparing Ang2-rtTA HFD to Ant2-rtTA ND for which the Wilcoxon test indicated nonsignificant differences (p = 0.20).

The statistical expert reviewed the Wilcoxon test results and advised basing the article’s results on Wilcoxon tests rather than t-tests. Therefore, the asterisk in Fig 3B for the comparison of rtTA to rtTA+cyto, and the asterisk in Fig 4D comparing Ang2-rtTA HFD to Ant2-rtTA ND should be disregarded, and the sentence in the Results discussing Fig 4D (“and fully abolished glucose stimulated insulin secretion (Fig. 4D, p<0.05).”) is not supported.

The Wilcoxon tests for Figs 4A and B in S3 File are results from repeated applications of the test conducted at each time point. The statistical reviewer noted that in order to conduct a thorough analysis, performing tests of interaction effects would be preferable. If the interaction effects are significant then the pairwise comparison at each time point could be presented. If the interaction effects are non-significant, the pairwise comparison at each time point would be excluded. The statistical reviewer also advised that a preferable approach to analyse the data in Figs 4A and B would be a repeated measures analysis, such as a mixed model that accounts for the interaction of groups by time.

In response to the concerns about the study power, the corresponding author stated that the sample size of 6 control vs 10 T2D autopsy samples from donor pancreases is of biological relevance for basic biomedical research findings derived from autopsy tissue as this comes from several biological replica and multiple technical replicas (at least 2–3) were analysed for each biological replica. They also commented on the rarity of the autopsy samples and stated that the statistical tests were approved by the ARC and Ethical committees.

During editorial follow-up the corresponding author also noted that the error bars in several figures in article [1] were presented as SD in error. The correct figures with error bars presented as SE (as stated in the captions in article [1]) are provided below and in S4–S6 Files. The corresponding author stated that these changes do not affect the interpretation of the data, conclusions or the reported results generated from individual values.

Individual-level underlying data from which charts in Figs 1B–1I, 2A, 2B, 2D, 2E, 2G–2M, 3B, 3D–3H, 4A–4H, S1A–S1D, S2A, S2F, S2H–S2J, and S3B–S3H were generated are provided here in S1 File. Original uncropped and unadjusted image files captured at the time of the experiment for all panels in Figs 2M, 3A, 3G–3H, S2B–S2E and S2G are also provided here in S2 File.

**Fig 1:**
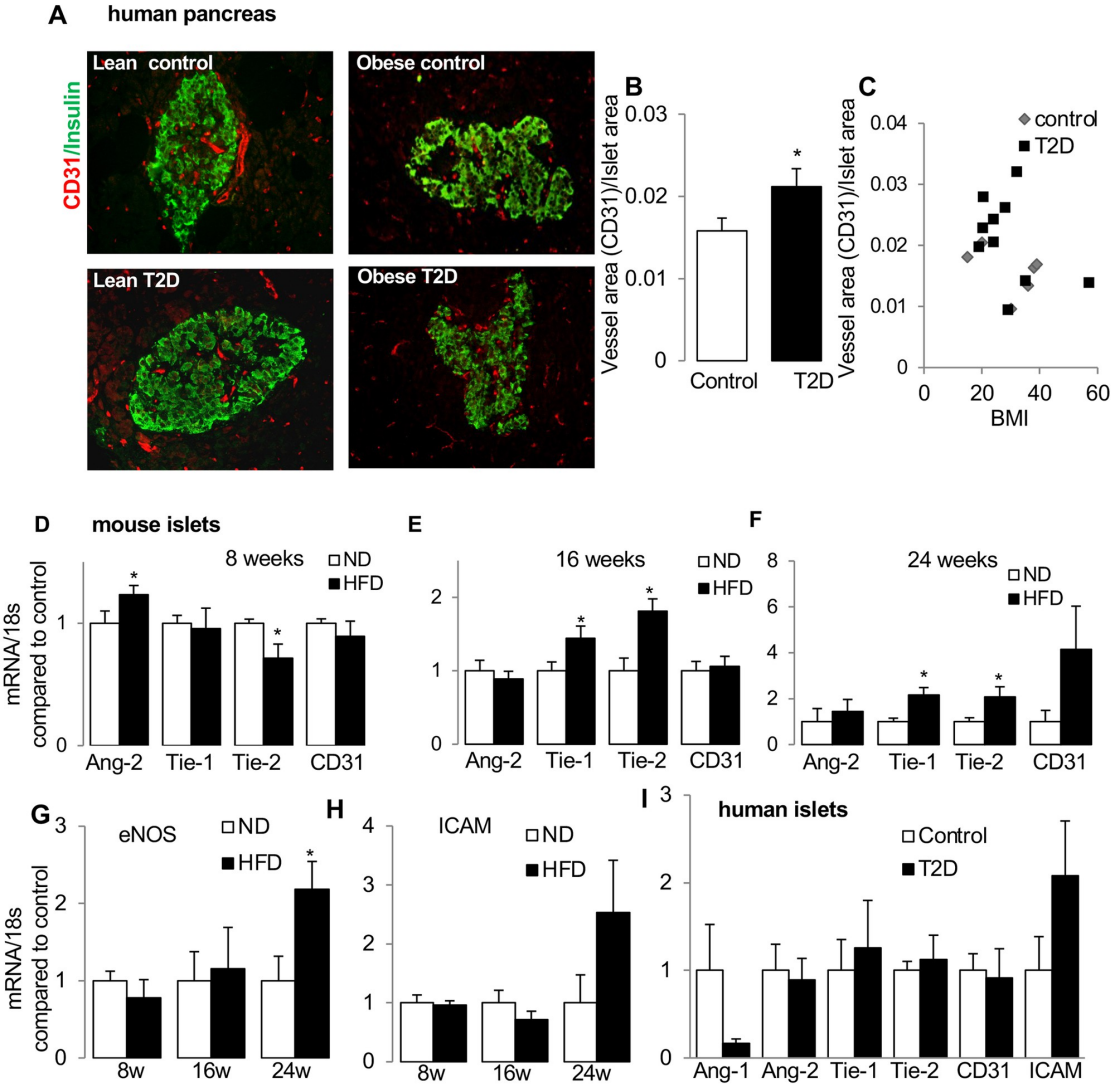
Islet vessel area increases in T2DM. (A) Representative images of pancreatic sections from non-diabetic controls and patients with T2D, immune-labelled for CD31 (red) and insulin (green). (B) Graphs show ratio of vessel area to islet area (control: n = 6; T2D: n = 10). (C) Plot shows no correlation of vessel density with BMI. (D-H) qPCR analysis of Ang-2, Tie-1, Tie-2, CD-31 from isolated mouse islets from C57BL/6 WT mice kept on normal diet (ND) or high-fat high-sucrose diet (HFD) for (D) 8 weeks (n = 4/group), (E) 16 weeks (n = 9/group) and (F) 24 weeks (n = 7/group), (G) of eNOS and (H) of ICAM-1. (I) qPCR analysis of Ang-1, Ang-2, Tie-1 and Tie-2 of isolated islets from non-diabetic (control, n = 8) and from patients with T2D (n = 7). *p<0.05, HFD vs ND or T2D vs. control.

**Fig 2:**
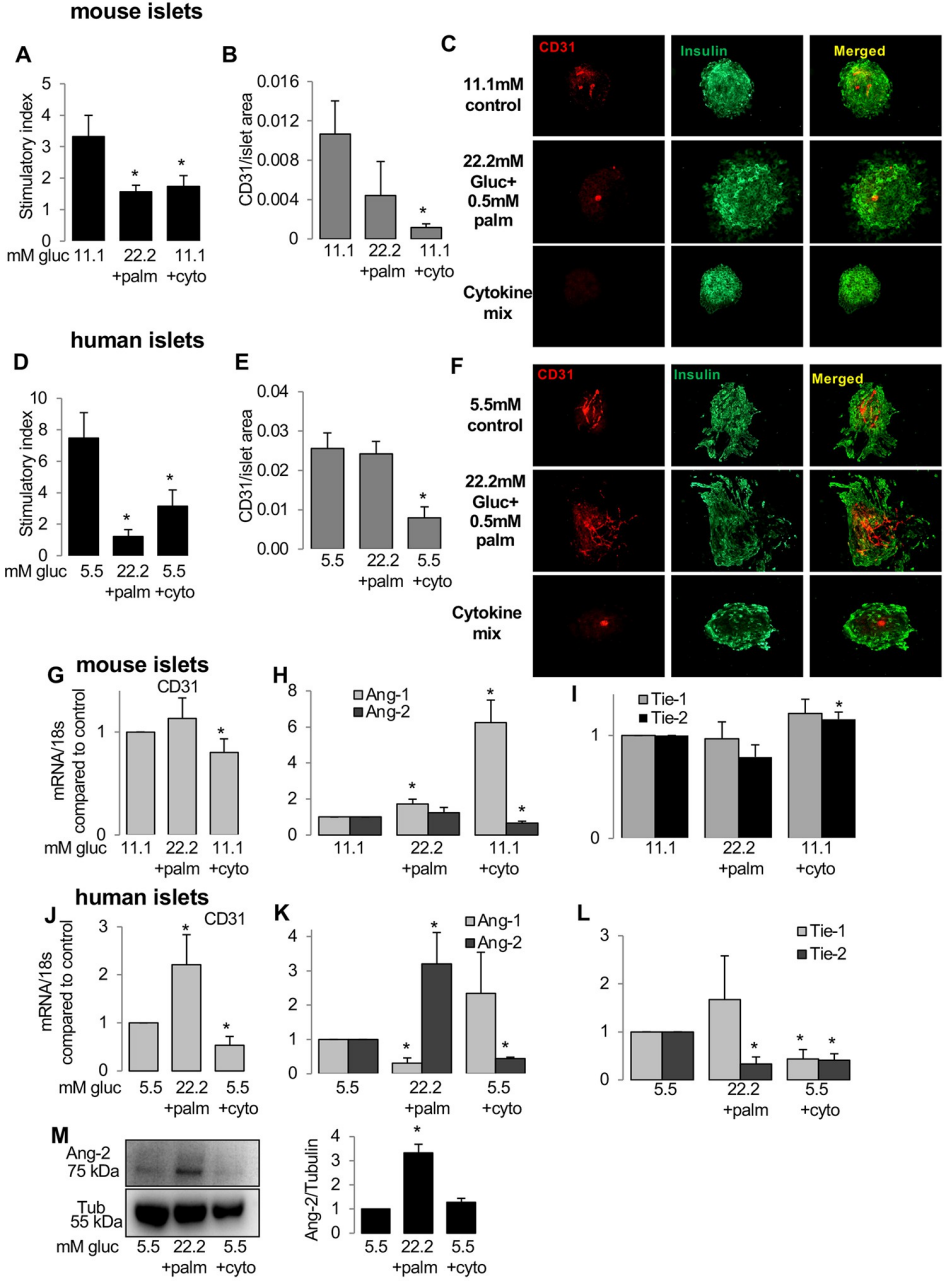
Ang/Tie expression in isolated islets correlates with changes in vessel area. Isolated WT mouse and human islets were cultured for 3 days in control condition (11.1 mM glucose for mouse or 5.5 mM for human) or treated with diabetic conditions of 22.2 mM glucose + 0.5 mM palmitic acid or mixture of cytokines: 2 ng/mL IL-1β, 1000 U/ml IFN-γ and TNF-α (cyto). (A,D) GSIS is shown by the stimulatory index assessed by 16.7/2.8 mM glucose stimulation. (B,C,E,F) Graph shows ratio of vessel area to islet area for mouse (B,C) and human (E,F) islets, fixed and immune-labelled for vessel (CD-31,red) and islet (insulin, green). (G-L) qPCR analysis of treated mouse and human islets for mouse CD-31 (G,J), Ang-1, -2 (H,K), Tie-1, -2 (I,L). All genes have been normalized to PPIA or 18s as housekeeping control. *p<0.05, treated vs. control 11.1 mM (mouse) or 5.5 mM (human). (M) Representative western blot from treated human islet lysates (left panel) and densitometric analyses of Ang-2 (right panel). Data are means +/-SE from 3–5 independent experiments from 3–5 different organ donors (human islets) or 3–5 independent mouse islet isolations.

**Fig 3:**
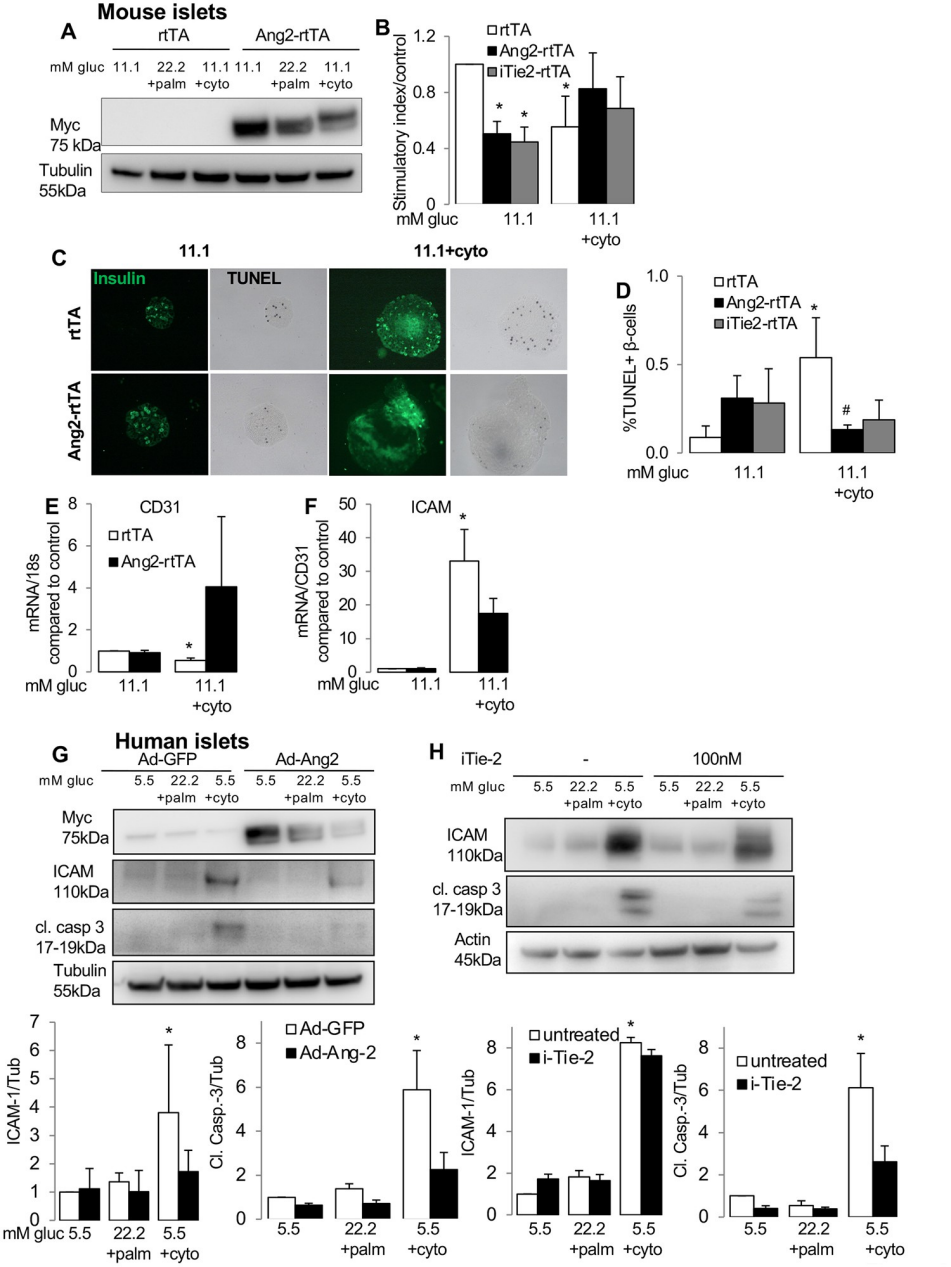
Ang-2 over-expression impairs islet function but protects from cytokine treatmentin isolated islets. Isolated islets from RIP-rtTA;tet-O-Ang-2 (Ang2-rtTA) and RIP-rtTA control (rtTA) mice were cultured for 3 days in presence of 10 μg/ml doxycycline to achieve Ang-2 overexpression. Mouse or human islets were cultured in 11.1 (mouse) or 5.5 mM glucose (human) or treated with diabetic conditions of 22.2 mM glucose + 0.5mM palmitic acid or mixture of cytokines: 2 ng/mL IL-1β, 1000 U/ml IFN-γ and TNF-α (cyto). (A) Western blot from treated mouse islets shows Ang-2 overexpression in islets by mycAng-2. (B) GSIS is shown by the stimulatory index assessed by 16.7/2.8 mM glucose stimulation and normalized to control. (C, D) Treated mouse islets fixed post-GSIS and apoptotic cells detected by double staining for TUNEL and insulin. Representative images from different treatments. (E,F) qPCR analysis for CD31 (E) and ICAM (F) from mouse islets overexpressing Ang-2. (G,H) Representative western blots (upper panel) and densitometric analyses of proteins (lower panels) showing myc-Ang-2, ICAM-1, cleaved caspase 3 and actin/tubulin as housekeeping control, in human islets overexpressing Ang-2 by Ad-Ang-2 or control Ad-GFP (G; MOI = 50) or treated with 100 nM Tie-2 inhibitor for 72h (H). Data are means +/-SE from 3–5 independent experiments from 3–5 different organ donors (human islets) or 3–5 independent mouse islet isolations. *p<0.05, treated vs. 11.1 mM glucose control, #p<0.05, Ang2-rtTA vs. rtTA.

**Fig 4:**
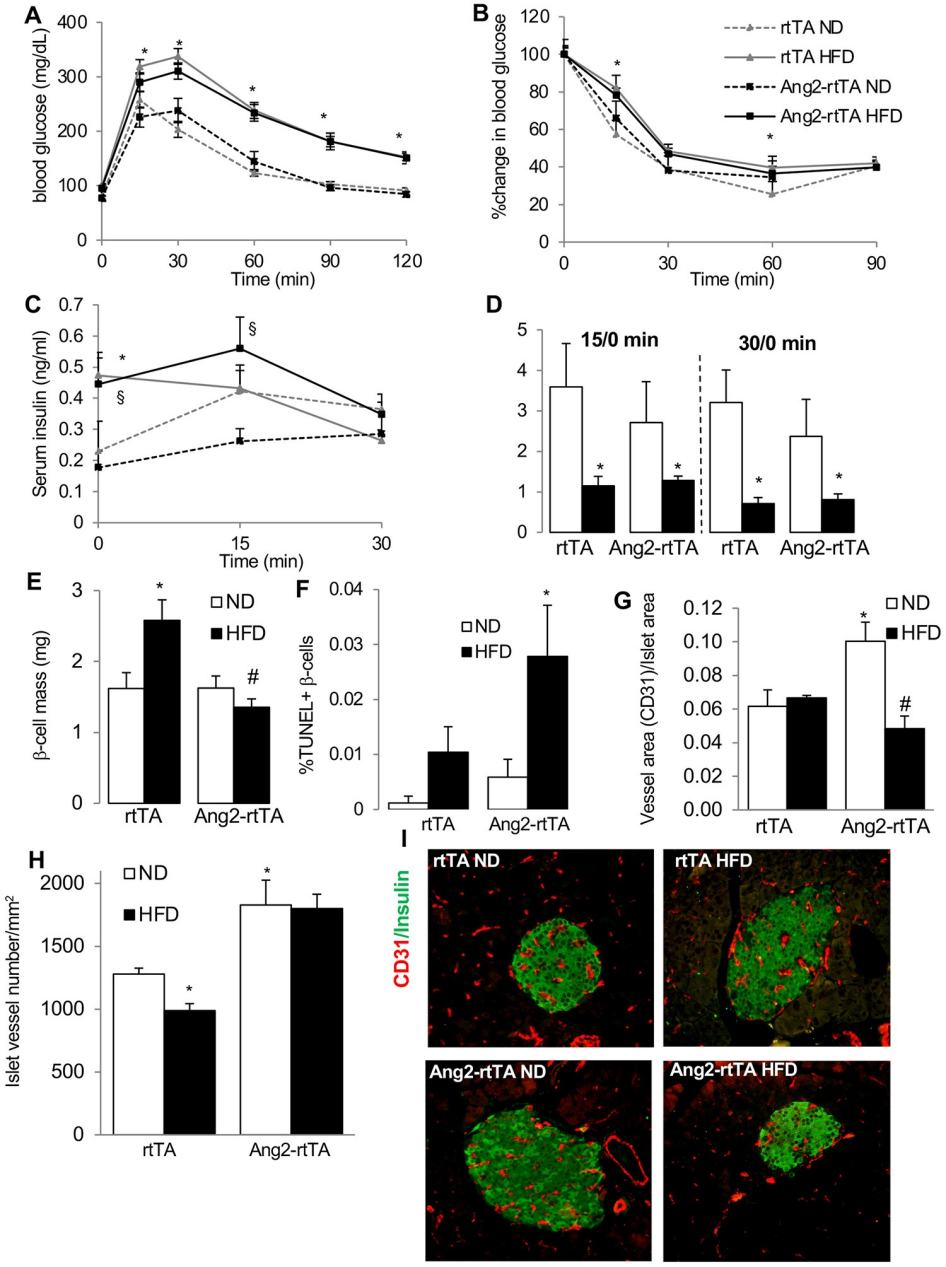
Ang-2 over-expression leads to islet hypovascularization and β-cell failure in response to HFD. β-cell specific overexpressing male Rip-rtTA;tet-O-Ang-2 (Ang2-rtTA) and Rip-rtTA (rtTA) kept on a normal diet (ND) or high-fat high-sucrose diet (HFD) received 1mg/ml doxycycline in drinking water for 16 weeks. (A,B) Blood glucose levels from intraperitoneal glucose (A) and insulin tolerance (B) tests performed at 16 weeks of HFD. (C,D) Glucose stimulated insulin secretion, showing levels of serum insulin and stimulatory index at 15 and 30 min after glucose injection. Data are means +/-SE from 4 independent experiments from n = 8 (ND) or n = 19 mice/group. (E-H) Pancreatic sections were analysed for (E) β-cell mass (F) apoptosis by double staining for TUNEL and insulin (10,000 β-cells/mouse, n = 6 mice/group) and (G) islet vessel area quantified by CD31/ Insulin co-staining (100 islets/mouse, n = 6 mice/group). (H) Islet vessel density from 3 animals per group represented as number of vessels per islet-mm2 (I) Representative images from CD31 (red)/Insulin (green) co-staining. *p<0.05 vs. rtTA ND, # p<0.05 vs. rtTA HFD, § p<0.05 vs. Ang2-rtTA ND.

Please note that reference 38 in article [1] was retracted [2] prior to the publication of article [1].

## Supporting information

S1 File Raw source data file with individual-level data in a single Excel file for each figure panel in a separate tab. (XLSX)

S2 File Uncropped western blots presented in article [1], labelled with the relevant panel. (PDF)

S3 File Non-parametric Wilcoxon Signed Rank Tests for the experiments in this article [1]. (XLSX)

S4 File Updated Supplementary Figure 1. (TIF)

S5 File Updated Supplementary Figure 2. (TIF)

S6 File Updated Supplementary Figure 3. (TIF)

## References

[1] Shah P, Lueschen N, Ardestani A, Oberholzer J, Olerud J, Carlsson P-O, et al. (2016) Angiopoetin-2 Signals Do Not Mediate the Hypervascularization of Islets in Type 2 Diabetes. PLoS ONE 11(9): e0161834. 10.1371/journal.pone.0161834 27617438 PMC5019443

[2] Ardestani A, Sauter NS, Paroni F, Dharmadhikari G, Cho J-H, Lupi R, et al. (2011) Neutralizing interleukin 1β (IL-1β) induces β-cell survival by maintaining PDX1 protein nuclear localization. J. Biol. Chem. 286(19) https://doi/org.10.1074/jbc.A110.210526 21393239 10.1074/jbc.M110.210526 PMC3089558

