# Supplementary figures and images for: Correction: Angiopoetin-2 Signals Do Not Mediate the Hypervascularization of Islets in Type 2 Diabetes
Source: PLoS One. 2023 Mar 2;18(3):e0282771. doi: 10.1371/journal.pone.0282771 (PMC9980724; doi:10.1371/journal.pone.0282771)

RAW WB images

Fig.2M

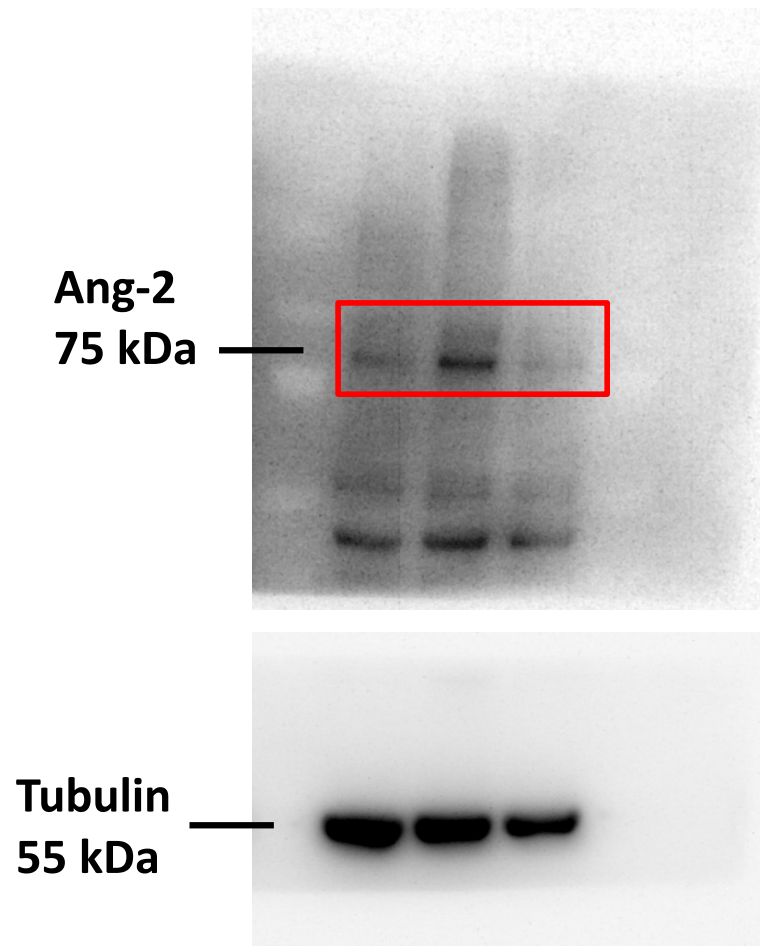

Fig.3A

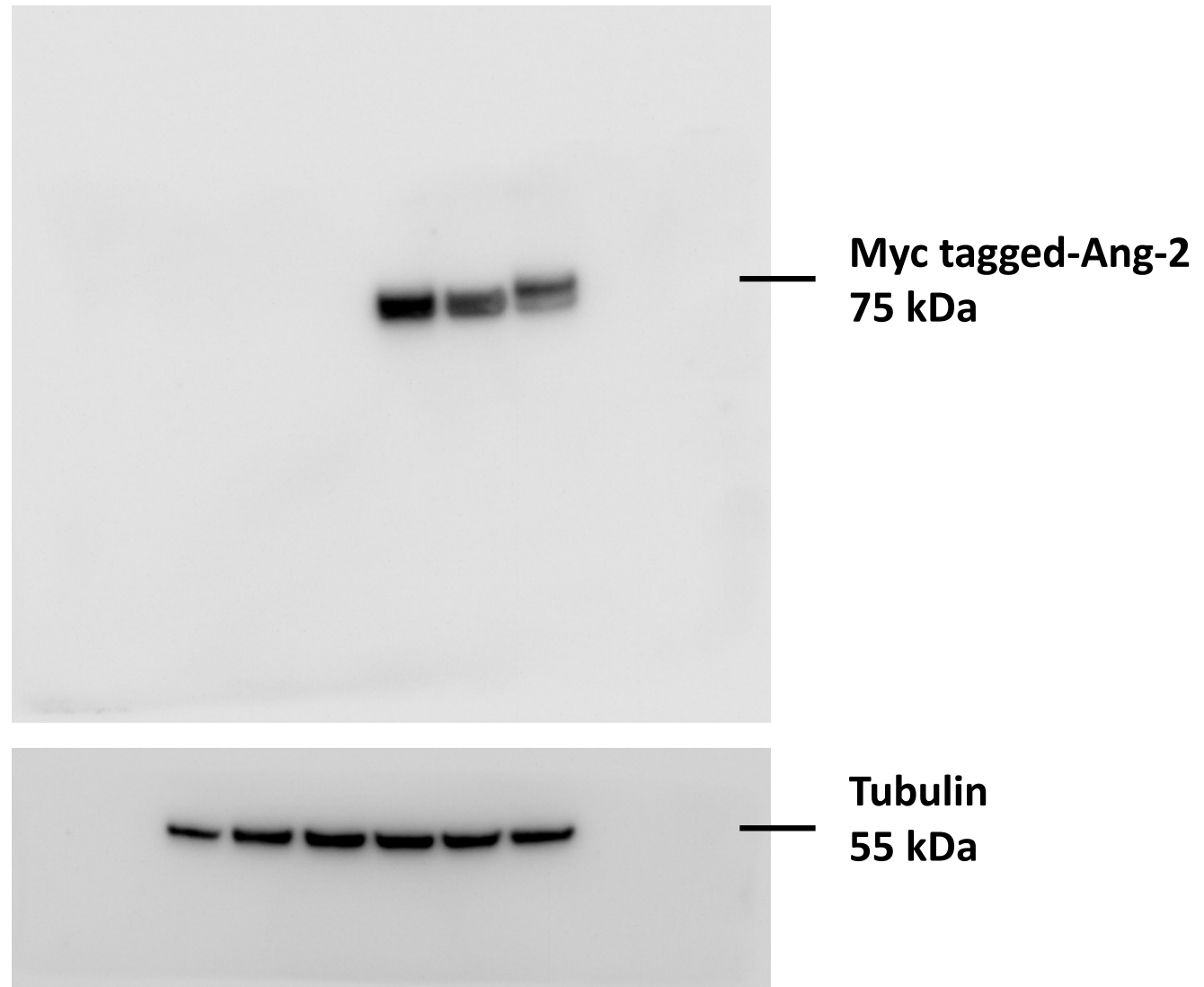

Fig.3G

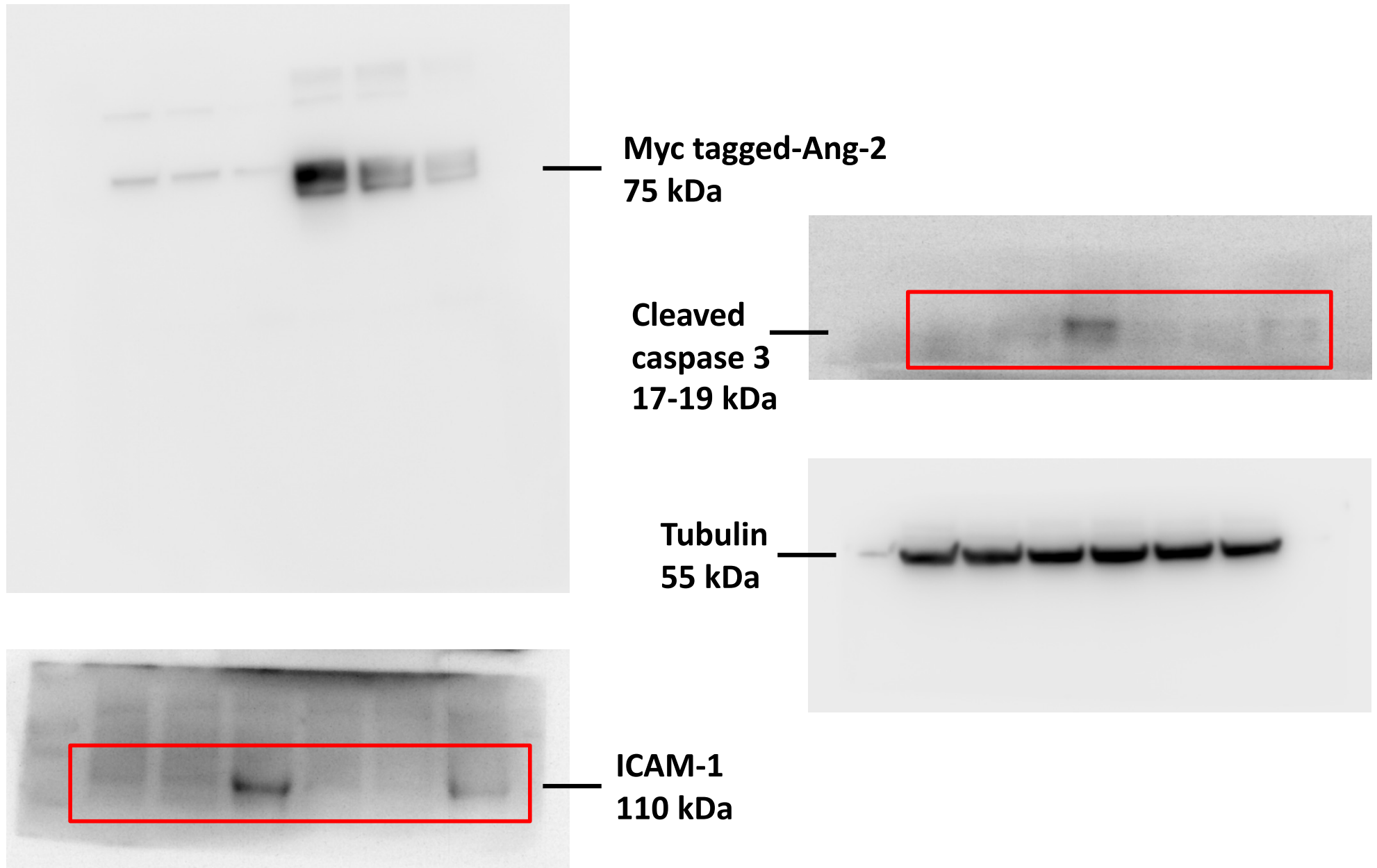

Fig.3H

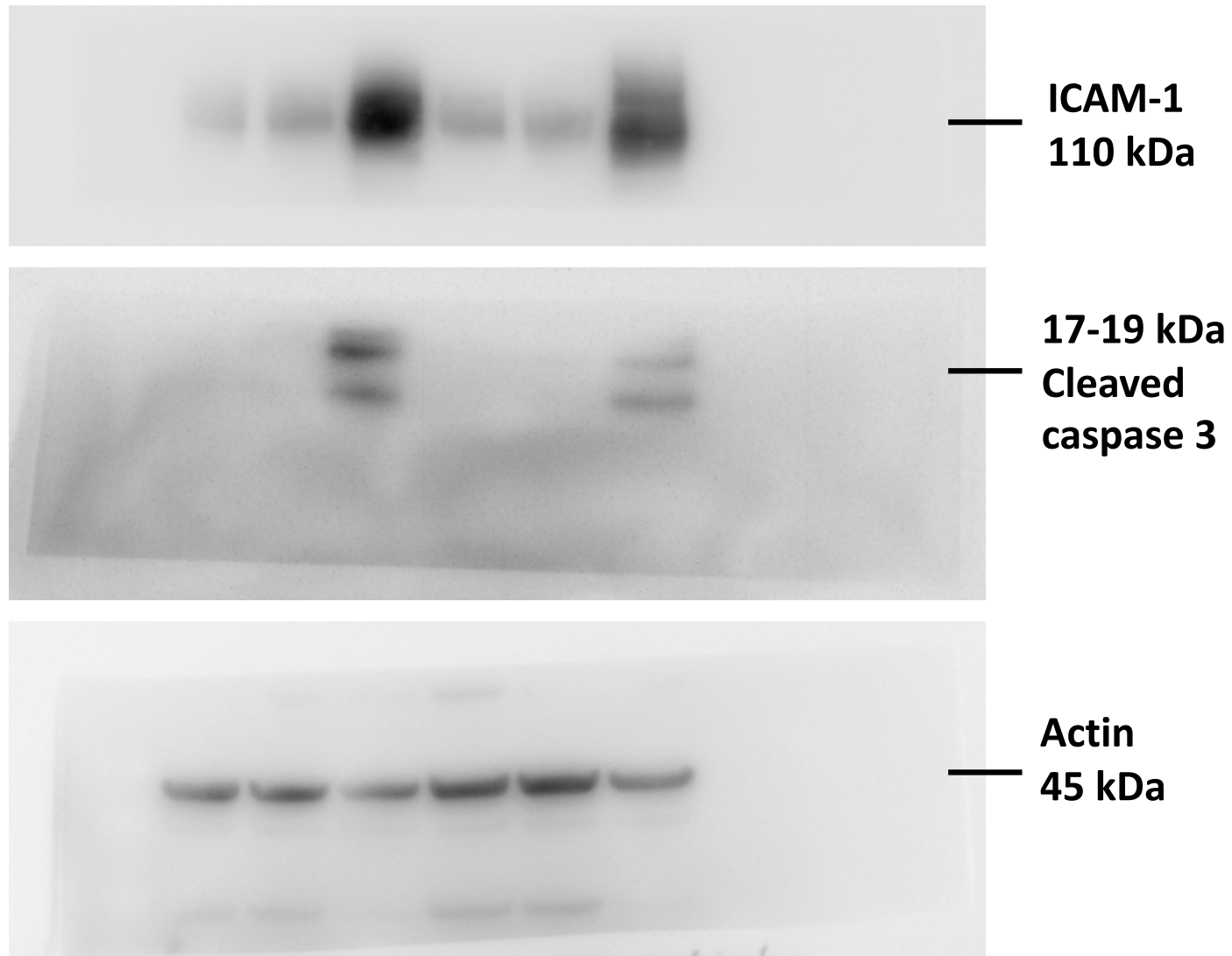

## Supp.Fig2B

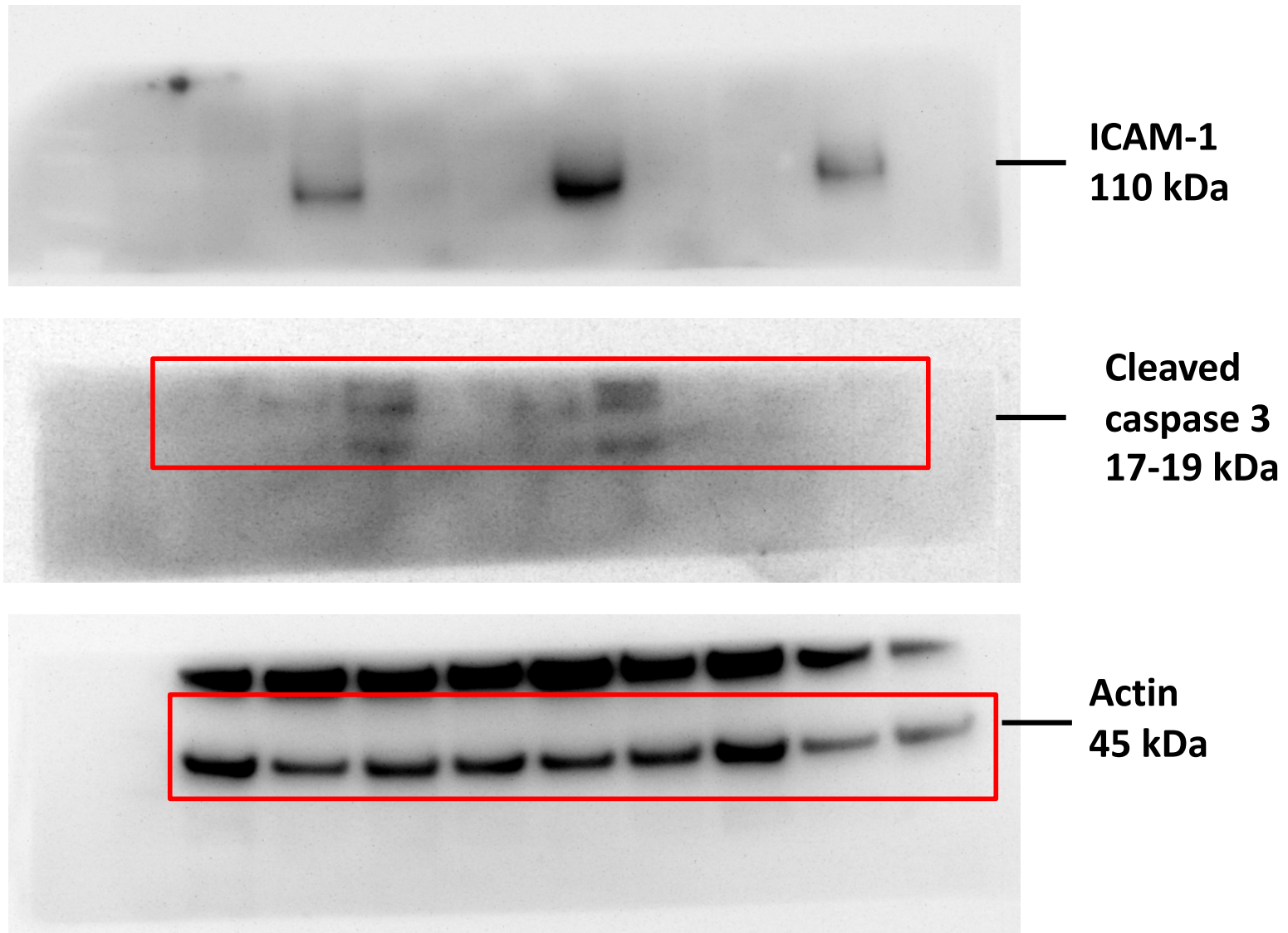

# Supp.Fig2C

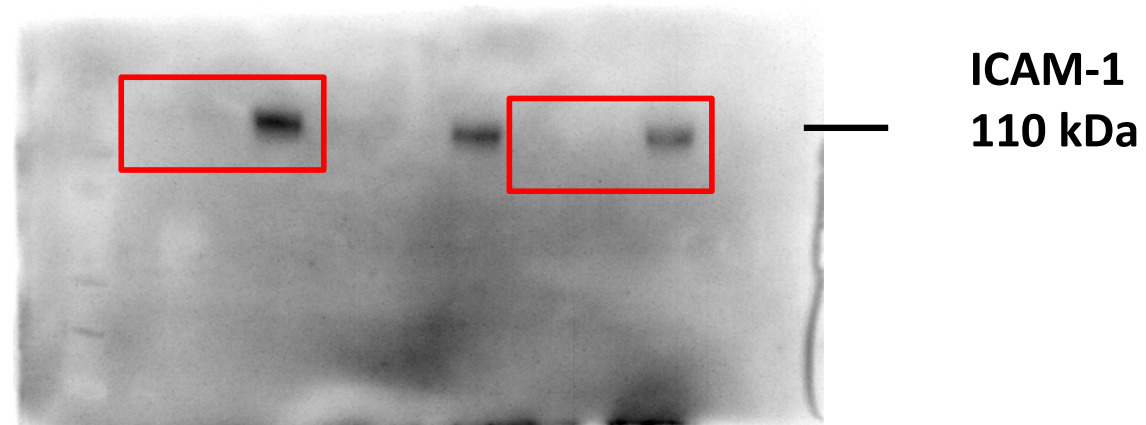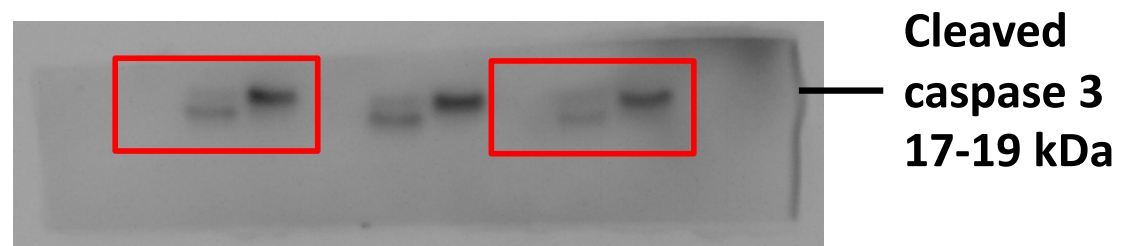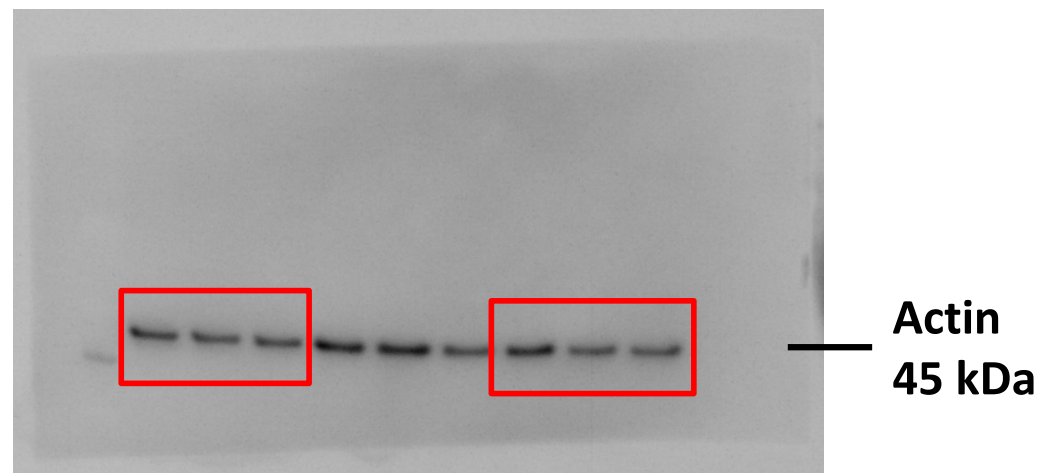

# Supp.Fig2D

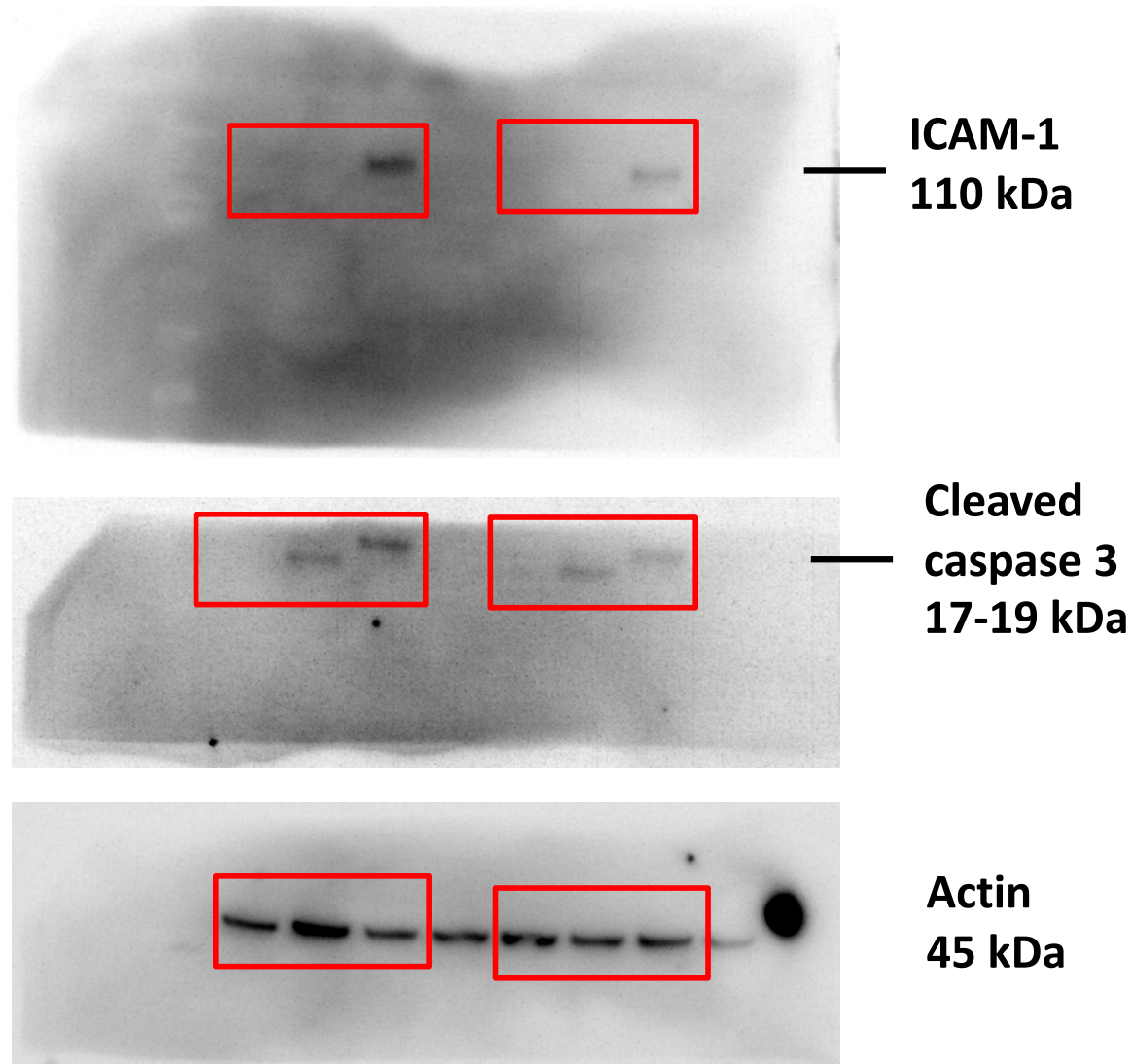

# Supp.Fig2E

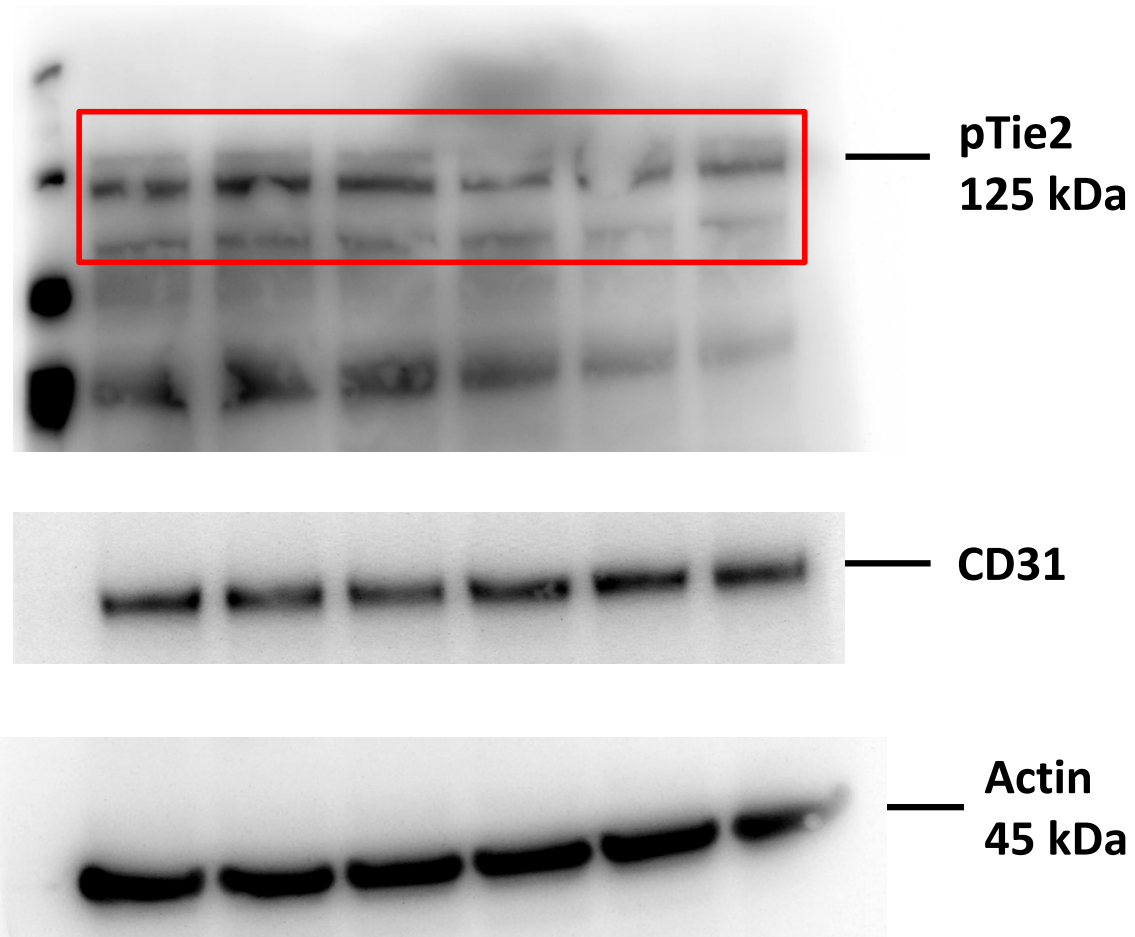

# Supp.Fig.2G

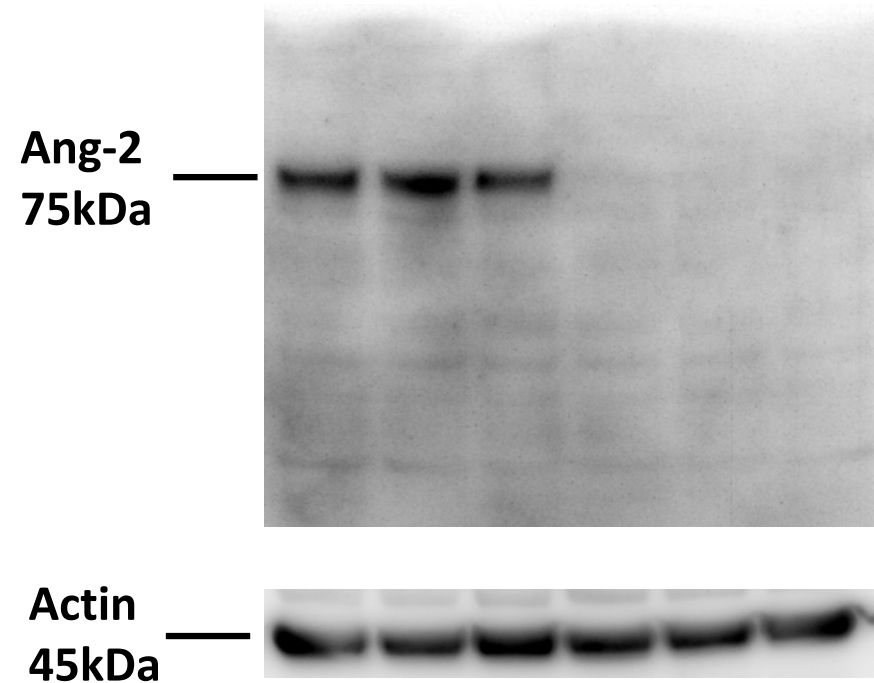

Supplement: S2 File — (PDF) [file pone.0282771.s002.pdf]

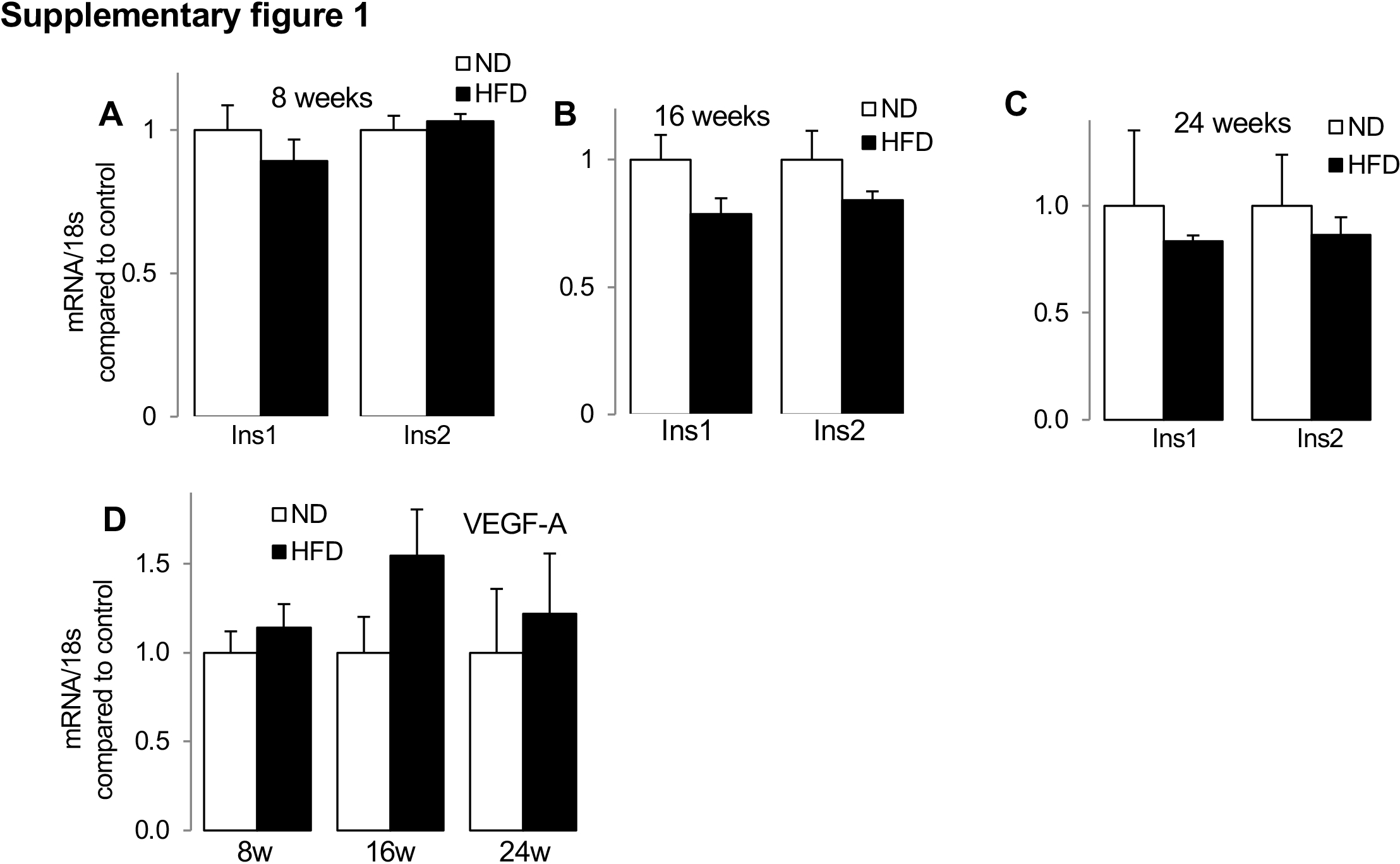

Supplement: S4 File — (TIF) [file pone.0282771.s004.tif]

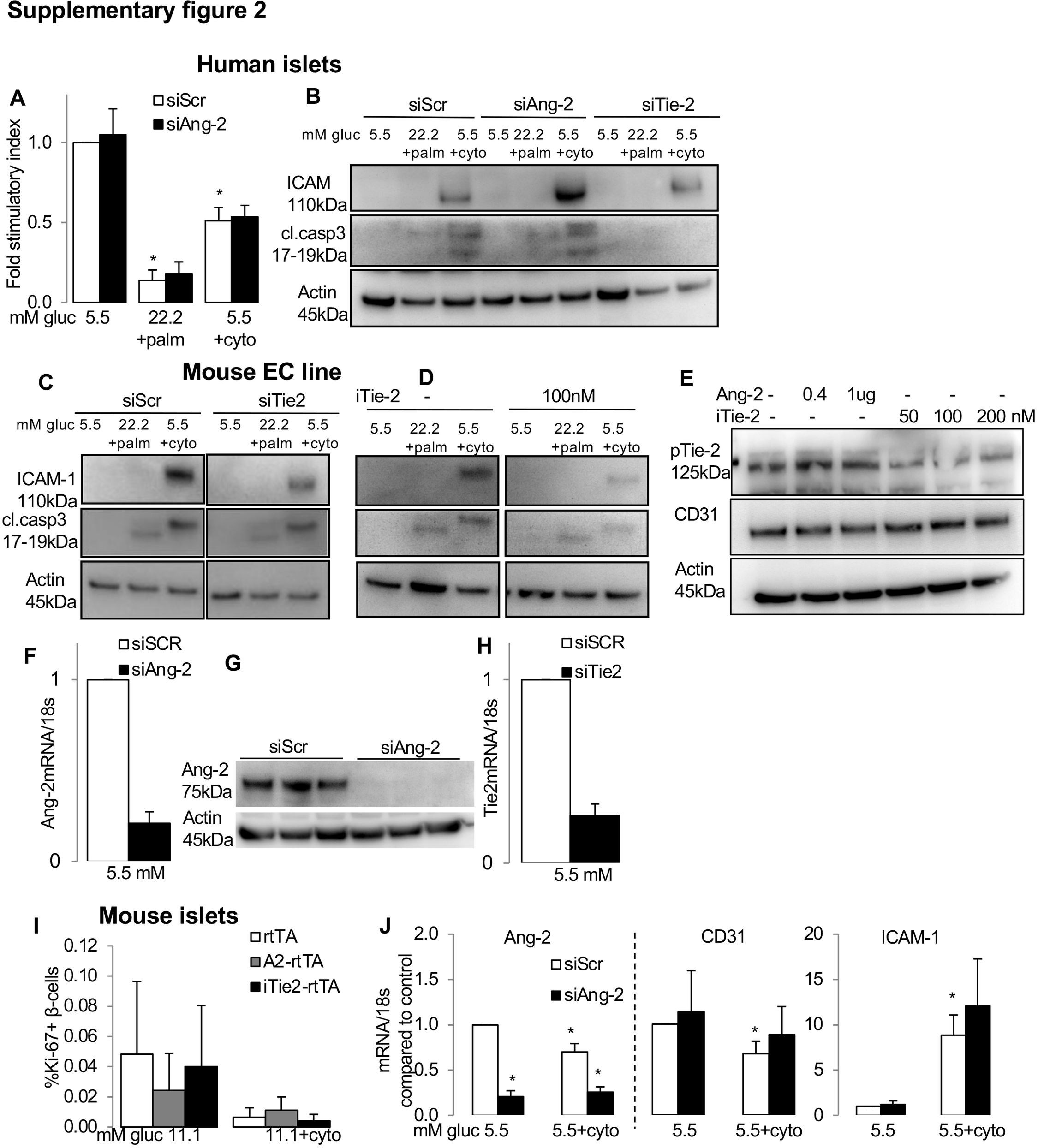

Supplement: S5 File — (TIF) [file pone.0282771.s005.tif]

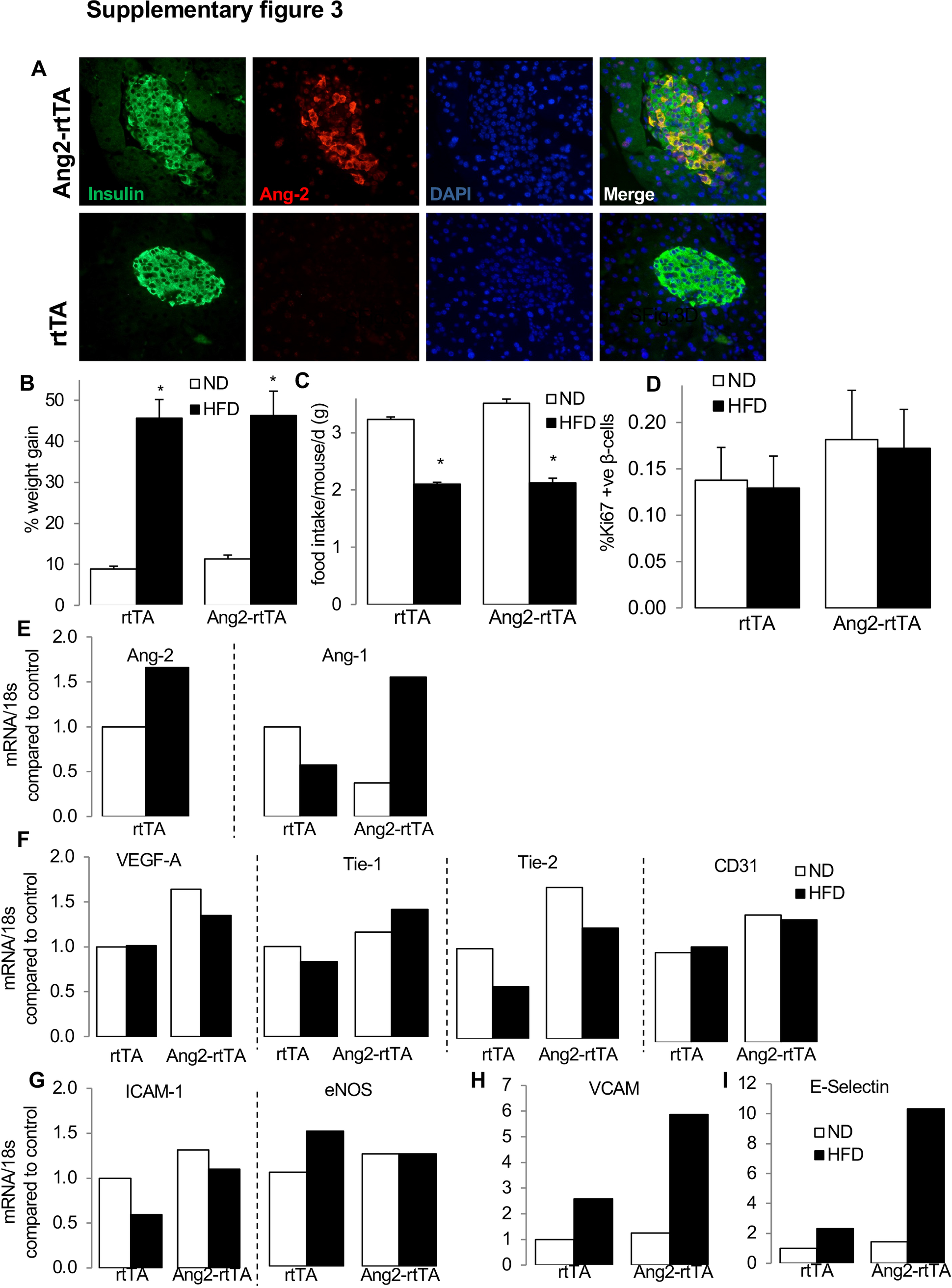

Supplement: S6 File — (TIF) [file pone.0282771.s006.tif]
